# The analysis of the gut microbiome during liver disease progression led to the identification of biomarkers for related mild cognitive impairment

**DOI:** 10.3389/fmicb.2025.1670512

**Published:** 2025-09-16

**Authors:** Lola Giner-Pérez, Juan-José Gallego, Carla Gimènez-Garzó, Daniela Batallas, Víctor H. Jarquín-Díaz, Franc Casanova-Ferrer, Alessandra Fiorillo, Amparo Urios, Jennifer N. Martínez-Medina, Adrià López-Gramaje, Yaiza M. Arenas, Desamparados Escudero-García, Salvador Benlloch, Alicia Salvador, Vicente Felipo, Sofia K. Forslund-Startceva, Gaspar Pérez Martínez, Carmina Montoliu

**Affiliations:** ^1^Laboratory of Lactic Acid Bacteria and Probiotics, Instituto de Agroquímica y Tecnología de Alimentos (IATA-CSIC), Valencia, Spain; ^2^Instituto de Investigación Sanitaria INCLIVA, Valencia, Spain; ^3^Departamento de Patología, Facultad de Medicina, Universidad de Valencia, Valencia, Spain; ^4^Laboratory of Neurobiology, Centro de Investigación Príncipe Felipe, Valencia, Spain; ^5^Laboratory of Social Cognitive Neuroscience, Department of Psychobiology and IDOCAL, Universidad de Valencia, Valencia, Spain; ^6^Experimental and Clinical Research Center, A Cooperation between the Max Delbrück Center for Molecular Medicine in the Helmholtz Association, Charité - Universitätsmedizin Berlin, Berlin, Germany; ^7^Charité – Universitätsmedizin Berlin, Corporate member of Freie Universität Berlin and Humboldt-Universität zu Berlin, Experimental and Clinical Research Center, Berlin, Germany; ^8^Max Delbrück Center for Molecular Medicine in the Helmholtz Association (MDC), Berlin, Germany; ^9^Servicio de Medicina Digestiva, Hospital Clínico Universitario de Valencia, Valencia, Spain; ^10^Departamento de Medicina, Universidad de Valencia, Valencia, Spain; ^11^Servicio de Medicina Digestiva, Hospital Arnau de Vilanova, Valencia, Spain; ^12^CIBERehd, Instituto de Salud Carlos III, Madrid, Spain; ^13^Department of Medicine and Surgery, Universidad Cardenal Herrera-CEU, CEU Universities, Valencia, Spain

**Keywords:** gut microbiome, biomarkers, minimal hepatic encephalopathy (MHE), metabolic dysfunction-associated steatotic liver disease (MASLD), cirrhosis, cognitive impairment, short-chain fatty acids (SCFA), neuroinflammation

## Abstract

**Introduction:**

Although it is well established that liver disease is associated with alterations in the gut microbiome (GM), the mechanisms linking these microbial changes to the progression of liver disease—and more critically, to its related cognitive impairment—remain poorly understood. Therefore, to define biomarkers for the early and advanced phases of these conditions, it is necessary to gain insight into changes in the GM throughout the evolution of the disease, particularly regarding the early onset of cognitive decline.

**Methods:**

The GM taxonomy and function profiles were defined, data were collected for dietary intake, fecal short-chain fatty acids (SCFA), cognitive status, quality of life and biochemical and immunological blood parameters of patients belonging to different stages of liver disease (MASLD and cirrhosis) and cognitive function.

**Results:**

This study showed: 1) the fibrosis stage severity (F1 to F4) in liver disease was associated with reduced GM diversity independently of cognitive status and with a decline in beneficial autochthonous bacteria; 2) *Streptococcus mutans* and *Allisonella histaminiformans* could serve as potential biomarkers for NAFLD-associated mild cognitive impairment; 3) bacterial metabolic functions involved in sugar degradation and the breakdown of tryptophan and glutamate were downregulated and linked to CXCL13 plasma levels and neuroinflammation; 4) correlations between SCFA concentrations disappeared with liver disease and cognitive impairment.

**Conclusion:**

In this context, maintaining a balanced production of fecal SCFA is more important than individual concentrations. The downregulation of specific microbial metabolic pathways, along with the presence of certain bacterial species, holds promise as early-stage biomarkers and highlights the potential of microbiome-targeted strategies for monitoring and managing liver-related cognitive impairment.

## Graphical Abstract

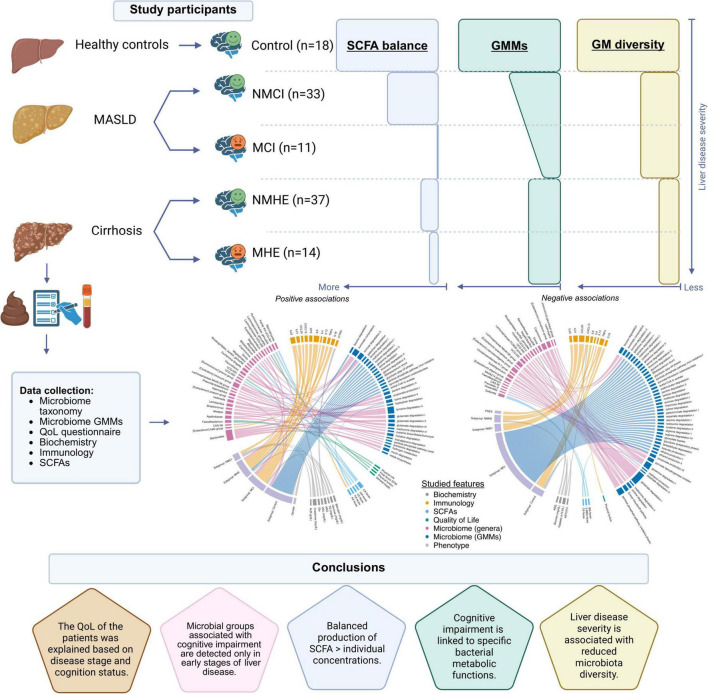

## 1 Introduction

The gut microbiome (GM) plays fundamental roles in the intestinal system by preventing the invasion of pathogenic microorganisms, metabolizing dietary elements into bioactive food components, and enhancing the immune system (Mills et al., 2019). The GM is altered in patients with liver cirrhosis (Bajaj, 2014; Bajaj et al., 2014) which may contribute to alterations in the immune system and cognition (Bajaj, 2014). Alterations in the gut-liver-brain axis seem to play a relevant role in the induction of minimal hepatic encephalopathy (MHE) (Bajaj, 2014), a complication of liver cirrhosis, characterized by mild cognitive and motor alterations (Nardone et al., 2016; Montagnese et al., 2022) that can progress to overt hepatic encephalopathy (HE) (Romero-Gómez et al., 2001).

Although it is considered that MHE appears in patients with liver disease only after reaching cirrhosis, our previous work has shown that patients with metabolic dysfunction-associated steatotic liver disease (MASLD) may already have mild cognitive impairment (MCI), with a lower prevalence than in cirrhotic patients (Felipo et al., 2012; Giménez-Garzó et al., 2021). MASLD is considered the hepatic manifestation of metabolic syndrome and has become a major cause of liver cirrhosis worldwide, with a global prevalence of 25% (Marchesini et al., 2003; Younossi et al., 2016).

Cirrhosis is associated with a notably altered gut microbial composition (Qin et al., 2014), as progression of dysbiosis has been associated with the worsening of liver disease conditions (Sydor et al., 2020). In cirrhotic and MASLD patients, the proportion of short-chain fatty acid (SCFA)-producing bacteria (*Lachnospiraceae, Ruminococcaceae*, and *Clostridium* group XIV) is decreased compared to *Enterobacteriaceae* and *Bacteroidaceae* (Bloom et al., 2021). Changes in the microbiota have also been observed in HE (Chen et al., 2021), furthermore, *Ruminococcus* and *Clostridium* group *XIV* are associated with better cognition in patients with MHE (Bajaj et al., 2019). Important evidence that HE is directly dependent on the GM is supported by the fact that rifaximin, a non-absorbable antibiotic, prevents the onset of HE and reverses cognitive impairment in a relevant proportion of patients with HE, in parallel with a reduction of endotoxemia and gut microbiome alteration (Kaji et al., 2017). Indeed, this antibiotic inhibits *Enterobacteriaceae* and streptococci and favors the proliferation of *Lachnospiraceae* and SCFA-producing clostridia in HE patients (Mancini et al., 2018). The administration of the non-absorbable antibiotic rifaximin to patients with HE evidenced the direct link between the gut microbiota and cognition (Kaji et al., 2017). Moreover, it also improves cognitive and motor function in 59% of cirrhotic patients with MHE and normalizes changes in the immune system and immunophenotype, despite the presence of non-responders—a characteristic feature of microbiome-associated diseases (Mangas-Losada et al., 2019).

However, the mechanisms linking the microbiota to the liver disease stages remain unknown. The characterization of microbial groups and functions associated with different stages of liver disease and cognitive function will help to understand the disease progression and provide keys to identify patients at higher risk of progression to more severe cognitive and hepatic states, and to design future interventions that could target the gut microbiome to prevent disease progression. Against this background, this study aimed to identify distinctive bacterial functional and taxonomic features of the GM in individuals at different stages of liver disease and with different levels of cognitive function to define potential biomarkers indicative of cognitive impairment associated with this disease, with a particular focus on the early stages of both conditions. These findings could improve early diagnosis and treatment of the disease, leading to an enhanced quality of life for patients.

## 2 Materials and methods

### 2.1 Study design and study population

This observational and cross-sectional study included 44 MASLD patients and 51 patients with liver cirrhosis, who were recruited from October 2020 to November 2022 in the outpatient clinics of Hospital Clínico and Hospital Arnau de Vilanova, Valencia, Spain. A group of 18 healthy subjects without liver disease, volunteers, of similar age to patients, were included as controls.

Diagnosis of MASLD or liver cirrhosis were based on clinical, biochemical, imaging features (mainly ultrasonographic data). As severity of MASLD relies mostly on fibrosis stage, patients with fibrosis stages F2 or higher (significant fibrosis) or F3 or higher (advanced fibrosis) are both considered high-risk patients. Fibrosis was assessed by liver biopsy when available or FibroScan parameters (>8 kPa for significant fibrosis and >12 kPa for advanced fibrosis). Therefore, in our study, subjects were grouped according to the progression liver disease: healthy control group (F0), MASLD patients without significant fibrosis (F0-F1), MASLD patients with significant fibrosis (F2-F3-F4) and cirrhosis group (F4).

Exclusion criteria for patients with liver cirrhosis were: overt HE, use of antibiotics (in the previous 6 weeks), alcohol intake (in the previous 6 months), established inflammatory, neurological or psychiatric disorders, recent use of drugs affecting cognitive function (in the previous 6 weeks), hepatocellular carcinoma, and liver-related complications (including new-onset ascites, variceal bleeding, or infection requiring antibiotics) in the previous 6 weeks, and patients with transjugular intrahepatic portosystemic shunts. For MASLD patients, exclusion criteria were recent use of drugs affecting cognitive function (in the previous 6 weeks), antibiotic use (in the previous 6 weeks), neurological or psychiatric disorders.

### 2.2 Ethics statement

All study participants were included after providing written informed consent. All the experiments were carried out in strict accordance with the principles outlined in the Declaration of Helsinki for human research. The study was approved by the Research Ethics Committees of the Hospital Clínico Universitario and Arnau de Vilanova Hospital of Valencia, Valencia, Spain (approval code: 2018/210; approval date: 2 March 2018; approval code: 2020/117; approval date: 26 November 2020).

Patients’ data were stored in an anonymized database, the confidentiality of which is guaranteed by the Organic Law on Data Protection 7/2021.

### 2.3 Diagnosis of MHE and MCI and psychometric tests

Sixteen cirrhotic patients were diagnosed with MHE using the PHES (Weissenborn et al., 2001) after adjusting for age and education level using Spanish normality tables^1^. Patients were classified as having MHE when PHES score was ≤ −4 points.

Twelve MASLD patients were classified as with MCI using a new sensitive score developed for patients with MASLD (Giménez-Garzó et al., 2021; Fiorillo et al., 2023). Patients were considered as having MCI when MCI score was < −5 (Fiorillo et al., 2023).

Healthy volunteers also undertook MCI score and PHES to discard any kind of MCI.

### 2.4 Sample collection

Blood ammonia was measured immediately after venous blood collection with the Ammonia Test Kit II for the PocketChem BA system (Arkray, Inc., Kyoto, Japan) according to the manufacturer’s instructions. Blood samples were centrifuged for 10 min at 1500 × *g*, and plasma and serum were immediately separated and kept at −80°C for subsequent cytokine analysis.

### 2.5 Detection of inflammatory markers

Plasma concentrations of IL6, IL18, IL23, IL21, IL13, CCL20, TNFα, CXCL13, IL22 and IL4 (R&D Systems, Minneapolis, MN, USA) were measured by ELISA according to the manufacturer’s instructions. High sensitivity kit was necessary for TNFα measurement.

Blood analytical data were collected for all participants. Controls and patients provided a stool sample for the study. Stool samples were flash-frozen in liquid nitrogen, and subsequently preserved at −80 °C until further analysis.

### 2.6 Fecal SCFA quantification

Fecal levels of SCFA (acetic acid, propionic acid, butyric acid, isobutyric acid, valeric acid, isovaleric acid, caproic acid, 2-Methylbutyric acid) were analyzed by HPLC-MS, as described in Mincheva et al. (2024).

### 2.7 Evaluation of health-related quality of life using the Short Form-36 health survey (SF-36)

Assessment of health-related quality of life (HRQoL) was conducted using the Spanish version of the Short Form-36 health survey (SF-36) (Alonso et al., 1995). The SF-36 relies on the patient’s perspective on health-related aspects, and it consists of 36 items related to different physical and mental health aspects, grouped into nine subscales: physical functioning, role-physical, bodily pain, general health, vitality, social functioning, role-emotional, mental health, transitional health (Bullinger, 1996), with scores ranging from 0 to 100, with higher scores indicating better HRQoL. SF-36 was corrected using a web application^2^, and global SF-36 score were calculated as average of multi-item scales.

### 2.8 Assessment of dietary profiles

All participants from disease groups (MASLD and cirrhosis) completed a validated Food Frequency Questionnaire (FFQ), which was used to calculate average daily intake (Schröder et al., 2011). Foods were classified in categories as specified in Supplementary Methods.

### 2.9 DNA extraction, sequence processing and bacterial profiling

Fecal DNA was extracted using an automated magnetic bead-assisted technique (Maxwell RSC Instrument with Maxwell RSC Pure Food GMO and authentication kit; Promega, Spain) according to the manufacturer’s protocol, further explained in Supplementary Methods. Amplicon sequencing of the V3-V4 hypervariable regions of the 16S gene was performed as previously reported (Illumina Inc, 2013). Based on the expected size of the 16S amplicon (459 bp), fragments between 400–428 bp (after trimming) were selected *in silico*. DADA2 (v1.18.0) pipeline was used to infer amplicon sequence variants (ASV) from the SILVA (v138. 1) database. Criteria for ASV filtering due to contaminants or low abundance are provided in the Supplementary Methods section. All analyses are based on non-rarefied data. All samples reached saturation in the rarefaction curves and the sequencing depth difference between samples was less than one order of magnitude (17,973– 82,629).

### 2.10 Statistical analyses

All statistical analyses were performed using R programming language (v4.1.2). A Principal Components Analysis (PCA) was performed to identify dietary patterns. Dunnett non-parametric tests and Wilcoxon rank sum exact tests subjected to Benjamini-Hochberg (BH) adjustments were used to compare patterns among groups.

Shannon, Chao1, and Fisher alpha diversity indices were compared among groups using Wilcoxon rank sum exact test and *P* values were adjusted for false discovery rate (FDR) using BH. Multivariate analysis was carried out using Principal Coordinates Analysis (PCoA) using Euclidean distances on the clr transformed data. Permutational analysis of variance (PERMANOVA) was performed for non-parametric multivariant statistical testing. No covariates or confounders were included in the diversity analyses.

MetadeconfoundR (v0.2.8) has been defined as “Covariate-sensitive analysis of cross-sectional high-dimensional data.” It operates in two stages, first using non-parametric tests, naive associations between omics features and metadata in cross-sectional data-sets are detected, in a second step, confounding effects between metadata associated to the same omics feature are detected and labelled through the use of nested *post hoc* model comparison tests (Birkner and Forslund-Startceva, 2024). Here, it was used to assess confounder-aware associations between ASV and GMM across metadata, detect confounded covariates, and investigate disease-related associations by comparing disease stages vs. control groups (MASLD vs. control, cirrhosis vs. control) and cognitive status (cognitive impairment vs. non-impairment). Dietary variables were not included in subsequent analyses based on the results of the PCA comparisons (See section “3.1 Inflammatory markers are systematically increased in patents with liver disease and cognitive impairment”). This analysis aimed to identify taxonomical, functional, and analytical parameter changes. Additionally, MetadeconfoundR was used for integrating data from six biological domains via a circos plot: Biochemistry, Immunology, SCFA, Quality of Life (QoL), Microbiome (Genera and GMM), and Phenotypic Data (Supplementary Methods).

A generalized linear mixed-effects model (GLMM) was used to assess the relationship between SCFA ratios and disease subgroups, with pairwise SCFA comparisons included as a random effect. Further details are provided in the Supplementary Methods. Moreover, logistic regression models were used as an exploratory approach to assess whether the abundance of selected taxa could discriminate between control and disease and between cognitively impaired and not cognitively impaired individuals. Model performance was evaluated using the area under the Receiver Operating Characteristic (ROC) curve (AUC), and sensitivity and specificity were calculated at the optimal threshold based on Youden’s Index.

Predicted the functionality of the gut microbiome was obtained using PICRUSt2 algorithm (Douglas et al., 2020) based on ASV counts. A detailed description of the used statistical methods and microbiome functionality prediction are provided in the Supplementary methods section. The scripts used for this work analysis can be accessed at https://github.com/lolapsgp/HE_humans_mainstudy.

## 3 Results

### 3.1 Inflammatory markers are systematically increased in patents with liver disease and cognitive impairment

**FIGURE 1:**
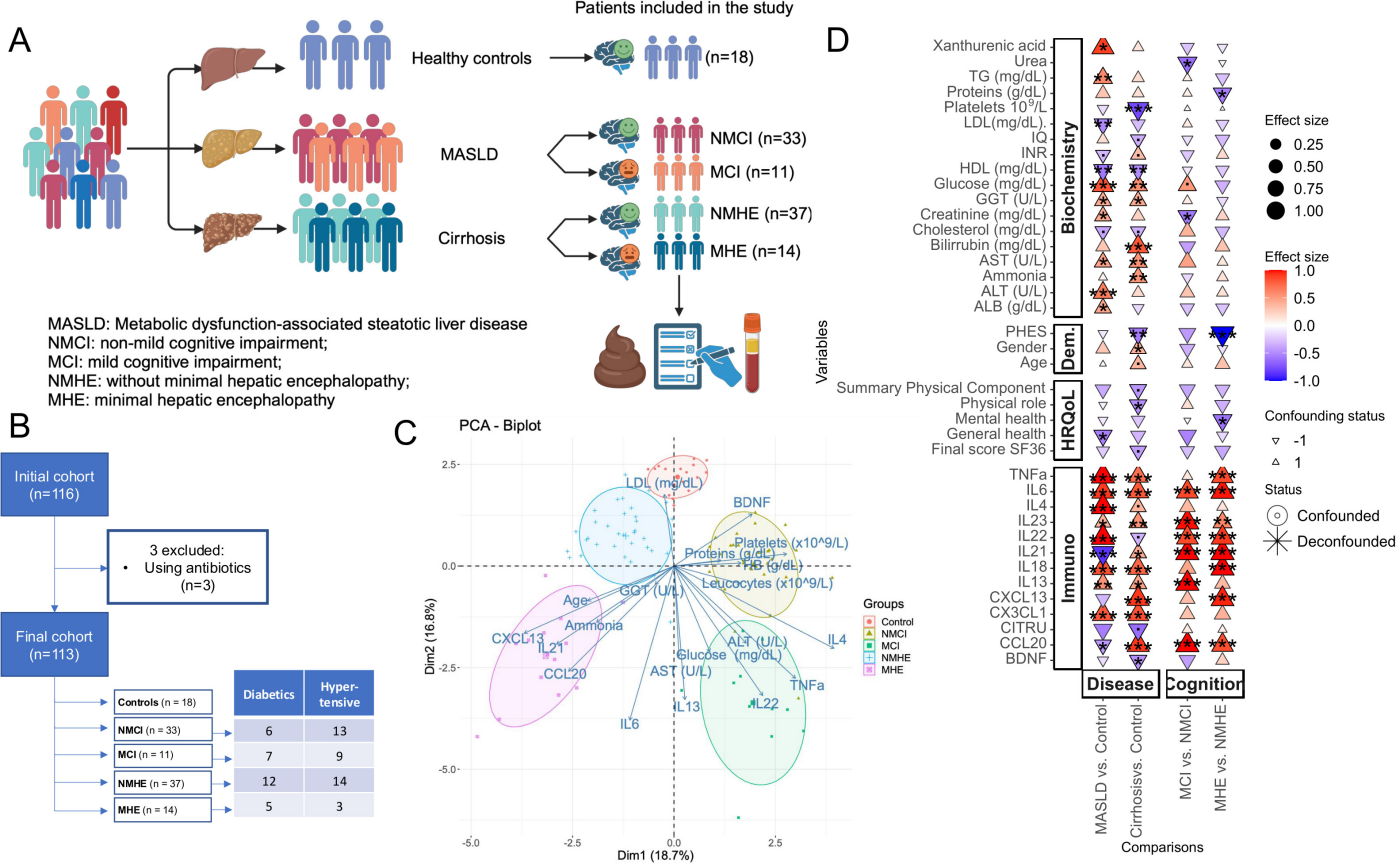
Patients’ data and experimental design. **(A)** Experimental design, including three disease groups (controls, patients with MASLD, and patients with cirrhosis) and five subgroups defined from the disease groups based on the presence or absence of mild cognitive impairment: controls, MASLD patients without or with mild cognitive impairment (NMCI, and MCI, respectively), and cirrhotic patients without or with minimal hepatic encephalopathy (NMHE and MHE, respectively). Created using BioRender. **(B)** Flow chart diagram. **(C)** Principal Components Analysis (PCA) including biochemical e immunological variables from which the vectors are represented as arrows in the plot. Each dot is one sample. **(D)** Biochemical, immunological, HRQoL and demographic (Dem) associations with disease stage (MASLD or cirrhosis) and cognitive status (with or without cognitive impairment), specifically within each disease stage. MCI (Mild Cognitive Impairment) denotes mild cognitive impairment in patients with MASLD, while MHE (Minimal Hepatic Encephalopathy) refers to patients with cirrhosis. The color scale and size illustrate the effect size, and the y-axis color scale on the left indicates the family to which each ASV belongs (indicated in the legend). Significance is denoted by black asterisks based on FDR-adjusted *p*-values. Significance levels: *P* < 0.1(.), *P* < 0.05*, *P* < 0.01**, *P* < 0.001***. Created with Biorender.com.

Sequencing of the 16S rRNA gene amplicon was performed from fecal samples of 116 subjects. Three patients were excluded due to antibiotic use. One subject in the control group was excluded from analyses involving adjustments by Psychometric Hepatic Encephalopathy Score (PHES) due to missing information. The analysis included a final cohort of 113 volunteers (and a single sample per volunteer). This final cohort had three groups that included 18 controls, 44 patients with MASLD, and 51 patients with cirrhosis, and the two patient groups were subdivided according to the cognitive profile. Thus, the study had a total of five subgroups defined as: 18 controls, 33 patients with MASLD without MCI (NMCI), 11 patients with MASLD and MCI (MCI), 37 cirrhotic patients without MHE (NMHE), and 14 patients with MHE (MHE) (Table 1). According to liver fibrosis stage, 20 MASLD patients (15 NMCI, 5 MCI) were included in the (F0-F1) group (without significant fibrosis) and 24 patients (18 NMCI, 6 MCI) showed significant liver fibrosis (F2-F4). The experimental design, patients’ flow chart, demographic and analytical parameters are shown in Figures 1A, B and Table 1.

**TABLE 1:** Demographic and clinical features of the patients and controls.

Variables	Control (*n* = 18)	MASLD patients		Cirrhosis patients	
		NMCI (*n* = 33)	MCI (*n* = 11)	NMHE (*n* = 37)	MHE (*n* = 14)
Age	58 (53.2, 61.5)	57 (50, 64)	60 (54, 65)	61 (57, 65)	65 (59.5, 69.2)*
Female gender n (%)	12 (66.7%)	10 (30.3%)	7 (63.6%)	9 (24.3)	5 (35.7%)
MASLD MCI Score^a^	0.0 (−2.0, 0.0)	−0.5 (−2.0, 1.0)	−7.5 (−10, −5)***/^ααα^	—	—
PHES^b^	0.0 (−0.5, 1.5)	–	–	−1 (−2, 0.0)*	−6.5 (−9, −5)***/^ααα^
**Fibrosis stage in MASLD patients^c^**					
F0–F1	–	15	5	–	–
F2–F4	–	18	6	–	–
Child-Pugh score (A/B/C)	–	–	–	36/1/0	5/6/3
**Biochemical variables**					
Ammonia (μM)	6 (6, 11)	6 (6, 14)	6 (6, 16.5)	19 (9.2, 47.7)**	33 (6.5, 63.5)*
Glucose (mg/dL)	88 (82.7, 90.7)	99 (92, 124)***	133 (127, 142)***/^αα^	105 (91, 124)***	92 (85.5, 104.7)
Creatinine (mg/dL)	0.74 (0.63, 0.76)	0.9 (0.77, 1.01)**	0.77 (0.69, 0.79)^αα^	0.79 (0.7, 0.85)	0.87 (0.68, 0.99)
Cholesterol (mg/dL)	196 (171.25, 203)	168 (153.5, 184)*	202 (167.25, 219.25)	172 (149, 207)	159 (120, 180)*
HDL (mg/dL)	59.5 (53, 67)	45 (41, 59)**	46 (38.2, 54.7)**	50 (46, 57)**	47.5 (38.2, 54)**
LDL (mg/dL)	122 (101, 126.7)	95 (73, 107)*	96 (75, 111)*	103 (82, 138)	95.5 (65.7, 115)*
TG (mg/dL)	93 (71.75, 99)	141 (104, 183)**	157 (102, 186.5)*	100 (81, 133)	89 (75.5, 104.75)
Proteins (mg/dL)	7.05 (6.92, 7.47)	7.3 (7, 7.5)	7.3 (7.15, 7.45)	7.5 (7, 7.9)	6.7 (6.5, 7.25)^αα^
ALB (g/dL)	4.3 (4.1, 4.3)	4.5 (4.3, 4.7)*	4.3 (4.2, 4.5)	4.2 (3.9, 4.5)	3.9 (3.2, 4.5)
Bilirrubin (mg/dL)	0.52 (0.41, 0.59)	0.66 (0.51, 1.24)*	0.55 (0.36, 0.64)^α^	0.97 (0.74, 1.25)***	1.2 (0.85, 1.87)***
AST (U/L)	25 (22.25, 28.25)	30 (23, 45)*	42 (36, 63)**/^α^	32 (26, 47)**	35.5 (28.7, 45.5)**
ALT (U/L)	22 (19.25, 26.5)	39 (25, 62)**	51 (45.5, 75)***	23 (18, 38)	28 (22.25, 35)
GGT (U/L)	34 (20.75, 57.25)	51 (35, 90)*	55 (41, 105.5)*	86 (34, 127)*	56.5 (30.25, 103.5)
Leucocytes (x10^9^/L)	6.665 (6.01, 7.85)	7.24 (5.9, 7.82)	7.7 (7.025, 8.465)	6.1 (5, 7.4)	6.17 (5.4125, 7.65)
HB (g/dL)	14.9 (13.975, 15.375)	15.4 (14.5, 16.3)	15.7 (14.1, 16.35)	14.1 (13.3, 15.4)	13.2 (12.5, 14.775)
Platelets (x10^9^/L)	233 (220.75, 270.75)	230 (204, 273)	236 (192.5, 257.5)	126 (97, 196)***	125.5 (91, 224.25)**
QI (%)	97.5 (92.75, 100)	100 (99, 100)	100 (96, 100)	95 (83, 100)	89 (72.25, 95)*
INR	1 (1, 1.0375)	1 (1, 1)	1 (0.92, 1)	1.05 (1, 1.12)	1.07 (1.03, 1.235)

Values are the median (1st quartile, 3rd quartile). Values significantly different from those in the controls are indicated by an asterisk (*) and from those in NMCI vs MCI or MHE vs. NMHE patients by α (*/α*p* < 0.05; **/αα*p* < 0.01; ***/ααα*p* < 0.001). MASLD, metabolic dysfunction-associated steatotic liver disease; NMCI, non-mild cognitive impairment; MCI, mild cognitive impairment; NMHE, patients without minimal hepatic encephalopathy; MHE, patients with minimal hepatic encephalopathy; HDL, high-density lipoprotein; LDL, low-density lipoprotein; TG, triglycerides; ALB, albumin; AST, aspartate transaminase; ALT, alanine transaminase; GGT, gamma-glutamyl transferase; HB, hemoglobin; QI, Quick index; INR, international normalized ratio.

^a^MASLD MCI score for diagnosis of MCI in MASLD patients.

^b^PHES (Psychometric Hepatic Encephalopathy Score) for diagnosis of MHE in cirrhotic patients.

^c^Fibrosis grade in MASLD patients from biopsy data when available (in 24 patients), or FibroScan parameters (20 patients).

For PCA analysis, age, several inflammatory parameters (IL6, IL13, IL21, IL22, IL4, TNFα, CCL20, CXCL13, BDNF), and biochemical parameters (ammonia, glucose, cholesterol, total proteins, aspartate transaminase, alanine transaminase, gamma-glutamyl transferase, hemoglobin levels, and leucocytes and platelets count) were included (Figure 1C). This analysis showed a clear separation between the different groups and it highlighted the importance of these variables in liver disease and cognitive impairment.

In order to identify changes in HRQoL, cognition, biochemical and immunological parameters specifically in the stage of liver disease or cognition, liver disease stage groups were compared to control group, and patients with cognitive impairment were compared to patients without it, within the liver disease group, respectively. Only variables that exhibited significant differences in at least one of the comparisons are displayed (Figure 1D). Noteworthy, immunological markers, which are typically associated with inflammation, are consistently increased in liver disease groups (MASLD and cirrhosis) when compared to controls, and in cognitive impairment groups (MCI and MHE) when compared to those without cognitive impairment (NMCI and NMHE). Moreover, data on age, sex, MASLD MCI and PHES scores, hepatic fibrosis grade in MASLD patients, liver disease severity in cirrhotic patients, and biochemical variables of the study groups are summarized in Table 1. No significant differences in age were observed between the groups, except for the age of patients with MHE, which was significantly higher than that of the control group.

Since diet is known to be a major community driver of the gut microbiota, FFQ were conducted to assess whether adjustments for dietary factors were necessary for subsequent analyses. Using PCA, four distinct dietary patterns were identified, each characterized by the consumption of a specific food group (Supplementary Figures 1A, B). No significant differences were observed among the five groups in relation to these dietary patterns (Supplementary Figure 1C); therefore, diet was not a factor to be further considered.

### 3.2 Liver fibrosis and cognition are associated with the microbiome, analytical parameters and HRQoL

Differences due to the fibrosis stages and cognition status in the main parameters analyzed in this work were tested with PERMANOVA. Fibrosis stages significantly explain taxonomic microbial composition and microbial functionality, explaining 4.6%, and 5% of the variance, respectively (Table 2), indicating that the similarity on the gut microbiota taxonomy composition is driven mainly by the level in liver tissue damage and not necessarily by the cognition status. Nevertheless, cognition had significant connection with microbiome functionality explaining 3.6% of variance (Table 2).

**TABLE 2:** PERMANOVA for gut taxonomic profiles (genus), functional profiles (GMM), biochemical parameters, immunological markers, and health-related quality of life (HRQoL) items from the SF-36 questionnaire.

Predictors and model terms	Taxonomic profiles				Functional profiles				Biochemical parameters				Immunological parameters				HRQoL			
	Df	F. Model	R2	Pr (> F)	Df	F. Model	R2	Pr (> F)	Df	F. Model	R2	Pr (> F)	Df	F. Model	R2	Pr (> F)	Df	F. Model	R2	Pr (> F)
**Fibrosis stage**	2	2.659	0.046	0.002**	2	3.083	0.05	0.01**	2	6.708	0.103	0.001***	2	51.821	0.313	0.001***	2	2.884	0.065	0.016*
Age	1	1.084	0.009	0.330	1	0.573	0.005	0.605	1	2.173	0.017	0.058	1	0.701	0.002	0.535	1	1.785	0.02	0.155
**Cognition category**	1	1.146	0.010	0.264	1	4.389	0.036	0.014*	1	0.451	0.003	0.829	1	69.183	0.209	0.001***	1	3.596	0.041	0.017*
Sex	1	1.100	0.009	0.306	1	0.379	0.003	0.81	1	0.652	0.005	0.685	1	0.164	0	0.943	1	2.306	0.026	0.085
Residual	105		0.901		105		0.854		105		0.808		105		0.317		75		0.849	
Total	111		1		111		1		111		1		111		1		81		1	

Significance levels are indicated as follows: *p ≤ 0.05, **p ≤ 0.01, and ***p ≤ 0.001. Underlined variables are described in the text. Significant variance predictors for each of the models are in bold.

Biochemical and immunological variables (Supplementary Table 1) were used to determine biochemical and immunological profiles of individuals (Table 1 and Supplementary Table 2). Fibrosis stage and cognition status significantly impacted the immunological profiles, accounting for 31.3% and 20.9% of the variability, respectively (Table 2). For biochemical profiles, fibrosis stage had a significant effect (*P* < 0.001), explaining 10.3% of the variability, while age had an effect close to significance (*p* = 0.058), explaining 1.7% of the variability (Table 2).

To determine how much of the patient’s quality of life changes due to the disease stage and the cognitive status, non-parametric Dunnett tests were conducted for pairwise comparisons between groups across the scales in the SF-36 questionnaire, revealing significant differences in 5 out of the 9 SF-36 questionnaire items (Supplementary Table 3). The PERMANOVA using HRQoL of the patients as response was also performed. The HRQoL was explained based on both the disease stage and the cognitive status, explaining 6.5% and 4.1% of variability, respectively (Table 2). Sex had a nearly significant effect, explaining 2,6% of variability.

The extensive data collection in this study allowed us to explore intricate interconnections between variables in the context of disease. This rendered subgroup classifications visualized as two circos plots illustrating the positive and negative interconnections between variables (Supplementary Figures 2A, B). This revealed a complex network of interdependencies, underscoring the importance of these variables in the context of disease. These plots highlight the importance of the positive and negative associations between the patients’ groups and inflammatory markers (immunology), cognition, the GMM, SCFA and bacterial genera. In the following sections, we explore specific correlations in detail, highlighting key associations and significant microbiome-related effects to derive meaningful conclusions for each domain.

#### 3.2.1 Severity of liver disease is associated with the diversity of the microbiome

**FIGURE 2:**
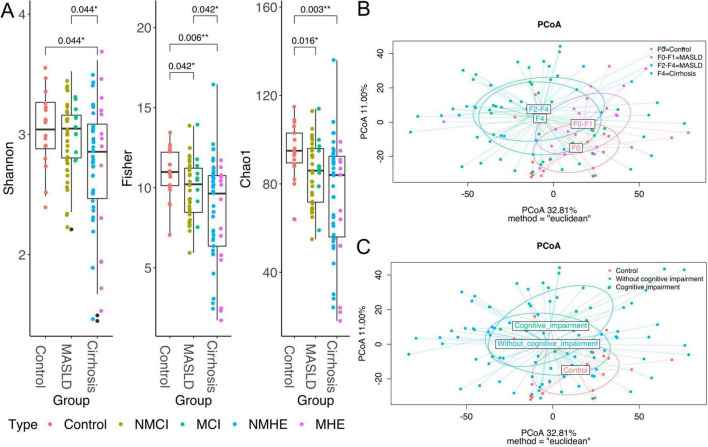
Diversity analyses. Boxplots representing Shannon, Fisher and Chao1 alpha diversity per disease group **(A)**, colors represent each subgroup within the group, specified in the legend. Plots of axes 1 and 2 of the Principal Coordinates Analysis (PCoA) of Euclidean distances used to compare the taxonomic diversity of gut microbiomes between populations, specifically, the clusters represent the fibrosis stages **(B)** and cognition status **(C)**. Taxa were agglomerated by genus for Alpha and Beta diversity analyses. The axis labels show the proportion of variance explained by each principal coordinate axis. *P* < 0.05*, *P* < 0.01**.

A significant decrease in α-diversity was observed, broadly reflecting the different stages of liver disease (Figure 2A). Microbial richness (Chao1 index) was significantly higher in healthy participants than in MASLD patients (Wilcoxon test, statistic = 561, p-adj = 0.016) and individuals with cirrhosis (Wilcoxon test, statistic = 698.5, p-adj = 0.003), with or without cognitive impairment. Similarly, diversity metrics, such as the Shannon and Fisher indices, exhibited a consistent decrease in patients with advanced liver damage stages, such as cirrhotic patients, compared to healthy participants. Notably, MASLD patients demonstrated significantly higher levels of Fisher diversity compared to those with cirrhosis (Wilcoxon test, statistic = 1415, p-adj = 0.042), suggesting a progressive decline in diversity from the control group to MASLD, and subsequently to cirrhosis (Figure 2A).

Neither richness nor diversity were different between the severity stages within MASLD (F0-F1 and F2-F4) (data not shown), between the NMCI group and those with MCI, as well as between cirrhotic patients without MHE and those with MHE (Supplementary Table 4), indicating that the cognitive status is not directly associated to microbiome alpha diversity.

Disease severity was associated with differences in the microbial composition (β-diversity dissimilarities). The group clusters clearly separate from the control cluster and expand with the increasing severity of the fibrosis stages (Figure 2B). Despite that PCoA could suggest a distance between control and cognitive impairment (Figure 2C), differences were not significant as seen before (Table 2).

### 3.3 Microbial groups associated with cognitive impairment are detected only in early stages of liver disease

**FIGURE 3:**
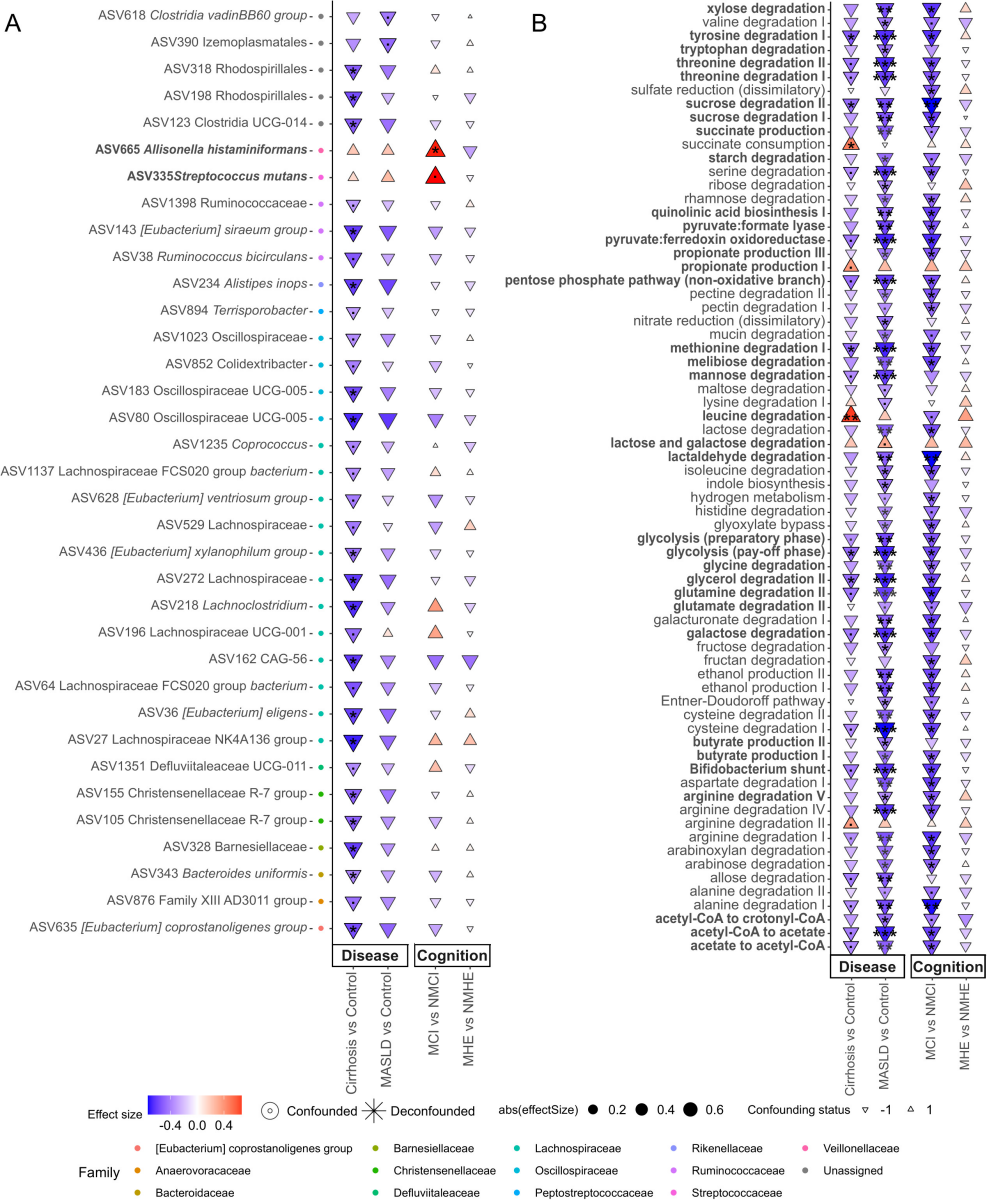
Features associated with disease stage and cognition status. Taxonomic **(A**, at ASV level) and functional **(B)** associations with disease stage (MASLD or cirrhosis) and cognitive status (with or without cognitive impairment), specifically within each disease stage. MCI (Mild Cognitive Impairment) denotes mild cognitive impairment in patients with MASLD, while MHE (Minimal Hepatic Encephalopathy) refers to patients with cirrhosis. The color scale and size illustrate the effect size, and the y-axis color scale on the left indicates the family to which each ASV belongs (indicated in the legend). Highlighted pathways (bold) are related to amino acid degradation, quinolinic acid synthesis, pathways related to carbon catabolite degradation and SCFA-related, and they will be mentioned in the Discussion section. Significance is denoted by black asterisks based on FDR-adjusted *p*-values. Significance levels: *P* < 0.1(.), *P* < 0.05*, *P* < 0.01**, *P* < 0.001***.

As shown before, gut microbiota composition is directly related to the severity of the disease, hence this section focuses on the finding of specific microbial markers associated with disease severity. Specific negative associations between bacterial taxa and MASLD and cirrhosis groups were identified using non-parametric tests that yielded a confounder-aware analysis of such associations. Figure 3A (disease effect panel) shows negative and positive associations of bacteria with the different conditions relative to healthy controls. Interestingly, cirrhosis and MASLD share the same negative associations, with most of them showing a higher effect size and significance (*P* < 0.01) in cirrhosis patients, suggesting that changes in abundance of specific disease-related bacteria may start during early progression of MASLD.

#### 3.3.1 *Streptococcus mutans* and *Allisonella histaminiformans* could serve as potential biomarkers for MCI

An important objective of this work was to determine the association of cognitive impairment with specific taxa or microbial metabolic functions in the different disease stages. In order to have groups with larger number of patients only two disease groups have been used for cognition analysis (MASLD or cirrhosis). No significant associations with cognitive impairment were found in cirrhotic patients. However, significant associations were found in MASLD patients (Figure 3A). MASLD patients with cognitive impairment were associated with *Allisonella histaminiformans* (Cliff delta size effect *d* = 0.53, FDR adjusted *P* < 0.05) and *Streptococcus mutans* (*d* = 0.55, *P* < 0.1) (Figure 3A). These two species showed positive association with the disease conditions, which could be due to the cognitively impaired patients.

Logistic regression was applied to the relative abundances of *St. mutans* and *A. histaminiformans* to evaluate their potential as biomarkers for MCI in patients with MASLD by determining the AUC. The model successfully discriminated between MCI and NMCI, achieving an AUC of 0.90 (95% CI: 0.71–1.00). The optimal threshold, determined using the Youden Index, yielded a sensitivity of 73% and specificity of 100% (Supplementary Figure 3). As these species appeared to be specific to the MCI group, a second model was constructed to evaluate their ability to distinguish between MCI patients and controls. This model achieved an AUC of 0.81 (95% CI: 0.72–1.00), with a sensitivity of 81% and specificity of 94% at the optimal threshold.

#### 3.3.2 There are greater changes in microbial pathways in MASLD compared to cirrhosis which are due to MCI patients

Changes in functional modules were similar in MASLD and cirrhosis compared to controls, but the strength and number of significant associations was higher at early stages of the disease (MASLD) (Figure 3B). In contrast, as described before, taxonomic differences were more pronounced in cirrhosis than in MASLD (Figure 3A). This tendency was also observed in the different fibrosis stages (Supplementary Figure 4). Such negative associations affected pathways related to amino acid degradation (tryptophan, tyrosine, threonine, methionine, glutamine, cysteine, serine, histidine, isoleucine, aspartate, alanine, valine, arginine and glutamate) and quinolinic acid synthesis. Also pathways related to carbon catabolite degradation (sucrose, maltose, lactose, fructan, starch, galactose, ribose, rhamnose, melibiose, mannose, glycolysis, pentose phosphate pathway and Bifidobacterium shunt), yielding metabolites like SCFA were significantly decreased (*P* < 0.01) as well as pyruvate related pathways leading to SCFA (pyruvate:formate lyase, pyruvate:ferredoxin oxidoreductase, acetaldehyde degradation, acetyl-CoA – acetate conversion, acetate-CoA to crotonyl-CoA), and propionate and butyrate production (Figure 3B). A few positive associations in cirrhosis compared to the control group were found, among which leucine degradation (*P* = 0.003, *d* = 0.66) and succinate consumption (*P* = 0.049, *d* = 0.48) showed the highest significance (Figure 3B). Interestingly, these GMM involved the production or degradation of metabolites previously associated to liver disease. Notably, the same microbial pathways were reduced in MASLD *vs.* healthy controls and in MCI *vs.* NMCI in the MASLD group, suggesting that the reduction in metabolic functions in MASLD may be due to reductions suffered by patients with MCI (Figure 3B and Supplementary Figure 2B).

### 3.4 Specific microbiome changes associated with HRQoL and analytical parameters

**FIGURE 4:**
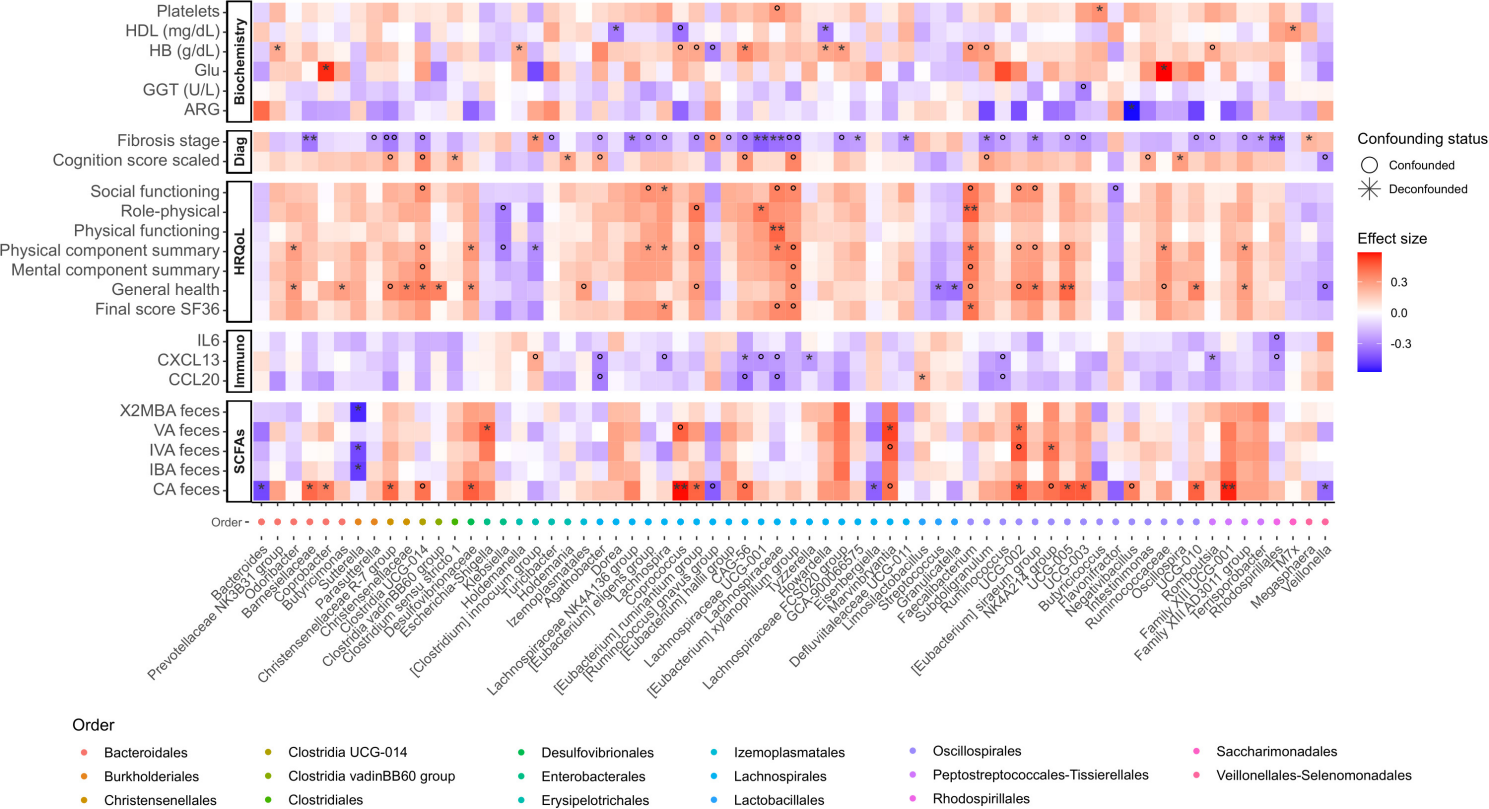
Heatmap displaying significant relationships between ASVs (Amplicon Sequence Variant) agglomerated by genus and meta-variables. The color scale on the heatmap represents the magnitude of the effect size, and the x-axis color scale on the left indicates the order to which each ASV belongs (indicated in the legend). Significance is denoted by black asterisks based on FDR-adjusted *p*-values, with grey circles representing associations that, although significant, are confounded. HDL, High-Density Lipoprotein; HB, hemoglobin; GGT, Gamma-Glutamyl Transferase; IL, Interleukin; CXCL13, Chemokine (C-X-C motif) ligand 13; CCL20, Chemokine (C-C motif) ligand 20; ARG, arginine; VA, valeric acid; IVA, isovaleric acid; IBA, isobutyric acid; CA, caproic acid; Glu, Glutamic acid; 2MBA: 2-Methylbutyric acid. Significance levels: *P* < 0.05*/°, *P* < 0.01**/°°.

Associations with taxonomic microbial composition and functional features were also tested for HRQoL. Although not all tests correlated with microbial genera, the direction of the significant associations (positive or negative) was consistent across tests. This consistency in the direction of associations was also reflected in the calculated final scores. Positive associations of this HRQoL final score included taxa from the *Faecalibacterium* genus, and *Lachnospiraceae* and *Oscillospiraceae* (UCG-002 and UCG-005) families. Negative associations included *Veillonellaceae* and *Streptococcaceae*, among others (Figure 4). Regarding GMM, succinate consumption and methionine degradation were negatively associated with the emotional role item. Methanol conversion was negatively associated with the general health item, and the module of lactose and galactose degradation was negatively associated with health transition (Supplementary Figure 5).

#### 3.4.1 CXCL13 is significantly associated to bacterial taxa and MHE

In light of the pivotal role of immune parameters (e.g., CXCL13, IL21, IL6, and CCL20) in the distinct microbial composition at early liver disease stages and in general with cognitive impairment (Figure 1C), we determined which specific bacterial taxa are related to these patterns. Multivariate analysis showed that CXCL13, IL6 and CCL20 are negatively associated with canonical SCFA producing genera such as *Lachnospira* and genera belonging to the *Lachnospiraceae* family, *Ruminococcus*, *Romboutsia* and *Rhodospirillales*. CXCL13 was positively associated with *[Clostridium] innocuum* group, identified as an emerging pathogen, and CCL20 and IL6 with *Limosilactobacillus*. Regarding IL21, no significant associations were found (Figure 4). Furthermore, CXCL13 also showed a high positive correlation with leucine degradation (Supplementary Figure 5). The negative association of CXCL13 to relevant bacterial taxa and functions point out to progressive immune dysregulation during liver damage.

### 3.5 SCFA imbalance is associated with disease stages

**FIGURE 5:**
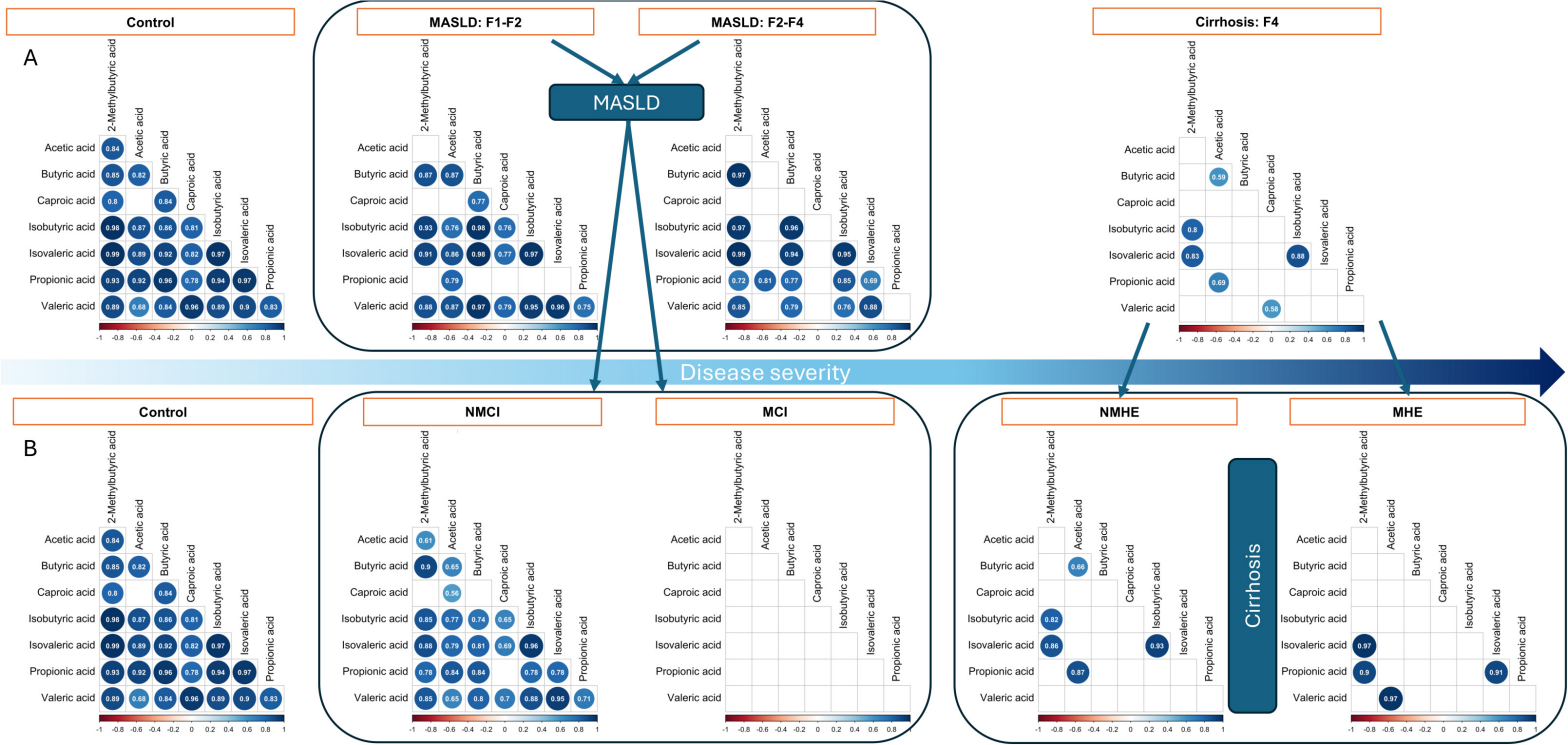
Relationship between fecal SCFA by fibrosis stage **(A)** and by subgroup **(B).** Non-significant coefficients are left blank, while significant correlations are shown by dots which size and color depend on the correlation size. MASLD, patients with metabolic dysfunction-associated steatotic liver disease; NMCI, and MCI, MASLD patients without or with mild cognitive impairment, respectively; NMHE and MHE, cirrhotic patients without or with minimal hepatic encephalopathy, respectively. Liver fibrosis stages are indicated as F1-F2, F3-F4, and F4.

Because liver damage has been shown to affect bacterial taxa and SCFA synthesis functions, the levels of several SCFA were measured in feces to ascertain whether local gut production and the observed deregulation could impact circulating levels. However, no statistically significant differences (Kruskal-Wallis, *p* > 0.05) were observed in the total fecal concentration of the different SCFA between the various stages of liver diseases (Supplementary Table 5 and Supplementary Figure 6). A notable observation was the shift in the correlation patterns of SCFA, which coincided with disease severity (Figure 5A). In healthy volunteers, we observed highly positive and significant associations between most of the SCFA, except for acetic acid-caproic acid (Spearman’s Rho > 0.6, *p* < 0.05). Of note, cognitive impairment had a strong impact on the correlations between the different SCFA within the MASLD group (Figure 5B), but in the cirrhosis group all samples displayed very low correlations and MHE was not very different from NMHE. The results obtained from these correlation analyses were further validated using a linear model that included the SCFA ratios as response variable and the disease subgroups as a fixed effect. The modeling approach, confirmed that SCFA proportions differ significantly among the groups [*F*(4,4815) = 11.47, *p* < 0.001]. In summary, these results showed a progressive SCFA imbalance associated with liver fibrosis and with cognition in the MASLD group.

## 4 Discussion

The GM plays a key role in the progression of liver disease, with alterations observed in both MASLD and cirrhosis (Qin et al., 2014; Chen et al., 2021). These microbial changes have also been linked to cognitive impairment associated with liver disease. Interestingly, the non-systemic antibiotic rifaximin, which modulates the GM, has been shown to restore cognitive function in MHE, suggesting a potential active role for the microbiome (Sidhu et al., 2011; Mangas-Losada et al., 2019). The present study aimed to investigate whether early-stage liver injury and its associated cognitive impairment correlate with progressive changes in the gut microbiome. This study is significant because it is the first to investigate the microbiome in the early stages of MASLD-related cognitive impairment. The study effectively demonstrated that liver injury leads to progressive changes in the gut microbiome. In particular, fibrosis stage was associated with microbial diversity and specific microbial changes, while cognition was found to be associated with microbial metabolic functions and a balanced production of SCFA.

Unlike previous studies (Bajaj et al., 2020; Yang et al., 2023), we found that disease progression was directly related to a decrease in GM α-diversity and showed an independent grouping of β-diversity patterns. As the disease progressed from MASLD to cirrhosis, a decline in potentially beneficial autochthonous bacteria from the *Lachnospiraceae, Ruminococcaceae, Clostridium* UCG-014, *Oscillospiraceae*, and *Christensenellaceae* families was observed. Similar patterns have been described previously in association with an increased proportions of potential pathogens (Ponziani et al., 2015; Kim et al., 2019; Zhang et al., 2022). However, in this work, the proportions of pathogens were not significant. Similarly, cognitive profiles were not significantly associated with changes in the microbiota, except for *A. histaminiformans* and *St. mutans* in MASLD patients with MCI. This is a novel finding in liver disease, though previous studies have reported an association between the oral pathogen *St. mutans* and cognitive impairment (Miyatani et al., 2015; Misaki et al., 2016). Our logistic models showed potential to distinguish MCI group from controls and from the NMCI group. However, given the sample size due to subgrouping and zero-inflation of the data, these results should be interpreted cautiously. These findings could justify further investigation of these potential biomarkers in larger or independent datasets.

Regarding cognition, MCI was associated with a lower abundance of bacterial species. In contrast, many GMMs, including leucine degradation, glutamate and glutamine pathways, and tryptophan metabolism, showed significant negative correlations with cognition. Some of these correlations had also been reported in MHE (Bajaj et al., 2021). Leucine degradation was associated with severe profiles of disease and cirrhosis, which is consistent with the lower levels of leucine and altered leucine metabolism observed in cirrhotic patients (Mullen et al., 1986). The gut’s glutamate and glutamine pathways are essential for communication between the gut microbiota and the brain, because some of the cognitive and motor impairments in HE result from changes in neurotransmitters activity on different glutamate receptors. These impairments can result from a buildup of glutamine, which leads to the swelling of brain cells (Albrecht and Dolińska, 2001; Cauli et al., 2006). Additionally, tryptophan-derived metabolites play a role in the development of neuroinflammatory and neurodegenerative diseases (Waclawiková and El Aidy, 2018; Clària et al., 2019). This study found that MCI in MASLD patients was linked to certain GMM, such as tryptophan breakdown, the production of quinolinic acid, the breakdown of glutamine and glutamate, various glycolytic pathways and related pathways involving acetyl-CoA and pyruvate, as well as several steps in butyrate production. Our study also showed that during the early stages of the disease, reductions of tryptophan metabolites, production of glutamine and glutamate, as well as reductions of SCFA by the gut microbiota, are very relevant milestones in the development of neuroinflammation. Strong changes in microbial pathways are observed in MASLD, particularly in MCI, as opposed to the small variation observed in the cirrhotic group. This suggests that profound microbiome changes occur in cirrhosis that no longer impact cognition. Our findings align with previous studies in rats, which also examined different stages of liver disease. These studies showed that mild liver damage leads to neuroinflammation and cognitive impairment. Such effects were prevented by early treatment with rifaximin (Balzano et al., 2021; Leone et al., 2022). These data suggest that rifaximin could be evaluated as a treatment in MASLD patients to prevent onset of mild cognitive and motor impairment.

Individual GM taxa showed important correlations with different variables, also with cognition. Advanced stages of fibrosis were negatively associated with a number of bacterial genera, mostly SCFA-producing *Firmicutes*, which could be connected to GMM such as carbon catabolite degradation pathways. Interestingly, the chemokine CXCL13 followed a similar pattern, showing an inverse correlation with SCFA-producing genera *Ruminococcus* and *Lachnospira.* In a previous work, we found that chemokine CXCL13 was higher in patients with MHE and correlated with PHES score (Mangas-Losada et al., 2017; Rubio et al., 2021). This chemokine promotes the expression of IL-22 and IL-17 (Marchesi et al., 2009), which in turn induce the expression of antimicrobial peptides (Liang et al., 2006), which could explain the remarkable impact on the microbial proportions in the gut ecosystem.

Some GM elements are also linked to biochemical and immunological factors in the volunteers. Hemoglobin was directly associated with *Coprococcus* and *Lachnospiraceae* bacteria, and platelet count was mainly linked to *Lachnospiraceae*. Trimethylamine N-oxide (TMAO) is produced in the gut and contributes to platelet hyperreactivity and enhanced thrombogenic potential. Serum TMAO levels were reported to increase in patients with MHE (Jiménez et al., 2010), which is consistent with the potential reduction of TMAO by *Lachnospiraceae* (Zhu et al., 2016).

Short-chain fatty acids (SCFA) have been extensively studied for their role in intestinal health. Functional analysis showed the relevance of the GMM related to butyrate production by gut microorganisms. These GMM correlated with butyric acid concentrations in samples, thus providing experimental support for the inferred microbial functions. Typically, individuals with liver disease have reduced levels of fecal SCFA (Dalile et al., 2019; Cao et al., 2023), and SCFA supplementation attenuates intestinal permeability in liver disease and it is a potential therapeutic method in numerous models of liver disease (Pohl et al., 2022). Furthermore, there is an inverse relationship between the prevalence of SCFA-producing bacteria and the incidence of overt HE (Bloom et al., 2021). SCFA may have a complex bidirectional regulatory interaction with the host. In this study, no significant differences in SCFA concentrations were observed between healthy controls and individuals with different stages of liver injury; however, there was a strong positive correlation between the concentrations of all the SCFA analyzed in healthy controls, except between acetic acid and caproic acid. The positive correlations between SCFA decreased with increasing liver severity, also within MASLD from F1-F2 to F3-F4. Most remarkably, differences in SCFA correlations were found between MCI and NMCI groups within MASLD; in cirrhosis, the correlations almost disappeared. This altered equilibrium of SCFA mirroring disease and cognitive impairment progressions can indicate that a balanced production of SCFA is a specific feature of the microbiome present in healthy individuals. The strong decrease in bacterial metabolic functions involved in sugar degradation (metabolic pathways leading to SCFA production) also supports our conclusion that, in the context of this work, a balanced SCFA production is a specific feature of the microbiome present in healthy individuals, particularly those with competent cognition.

This study encountered some limitations. First, dietary influence on gut microbiome changes of participants. To address this limitation, participants completed a food frequency questionnaire. The food questionnaires were used to identify dietary patterns to compare among and between groups, since no differences were found, diet was not included as a major factor in the subsequent analyses. Another limitation was only relying on 16S data for microbial profiling, which has limitations in terms of taxonomic resolution and functional pathways; however, this tool remains effective in capturing essential microbial patterns. Moreover, this cross-sectional study could not establish causal relationships, underscoring the need for further mechanistic research. These limitations must be addressed in future research. Specifically, larger human cohort studies using whole genome sequencing, as well as animal models and cultures, are necessary to identify cause-and-effect relationships.

This study showed that liver disease progression is reflected in microbiome diversity, regardless of cognitive status. GM diversity decreases with liver disease progression, as does the presence of potentially beneficial autochthonous bacteria. Cognitive impairment was associated with decreased bacterial metabolic functions involving tryptophan, glutamate, and glutamine degradation. This suggests that metabolic deficiencies in the microbiome may contribute to sustained neuroinflammation during MASLD. These microbial pathways were also significantly associated with CXCL13 production. *S. mutans* and *A. histaminiformans* were identified as potential biomarkers for distinguishing MASLD patients with MCI from those without cognitive impairment and from the control group in the early stages of the disease. Our study shows that balanced production of different SCFAs in the gut is more important than individual concentrations and is an excellent indicator of a balanced microbiome. These findings improve our understanding of the microbiome’s intricate interactions in liver disease, particularly its association with cognition. They also reveal an unexplored area of gut microbiota research and establish the grounds for monitoring disease progression to support patient care in liver disease and impaired cognition.

## Data Availability

The datasets presented in this study are publicly available. This data can be found here: https://www.ncbi.nlm.nih.gov/, accession PRJNA1242276.

## Footnotes

1 www.redeh.org/TEST_phes.htm accessed on December 2022

2
https://orthopowertools.com/SF36
