# Supplementary material for: The analysis of the gut microbiome during liver disease progression led to the identification of biomarkers for related mild cognitive impairment
Source: Front Microbiol. 2025 Sep 16;16:1670512. doi: 10.3389/fmicb.2025.1670512 (PMC12481516; doi:10.3389/fmicb.2025.1670512)
Supplement: Supplementary file 1 [file Supplementary_file_1.docx]

**SUPPLEMENTARY MATERIAL**

**The analysis of the gut microbiome during liver disease progression led to the identification of biomarkers for related mild cognitive impairment.**

Lola Giner-Pérez^a^, Juan-José Gallego^a^, Carla Gimènez-Garzó, Daniela Batallas, Víctor H. Jarquín-Díaz, Franc Casanova-Ferrer, Alessandra Fiorillo, Amparo Urios, Jennifer N. Martínez-Medina, Adrià López-Gramaje, Yaiza M. Arenas, Desamparados Escudero-García, Salvador Benlloch, Alicia Salvador, Vicente Felipo, Sofia K. Forslund-Startceva, Gaspar Pérez Martínez*, and Carmina Montoliu*

^a^These authors contributed equally to this work.

*Correspondence: Gaspar Pérez Martínez ([gaspar.perez@iata.csic.es](mailto:gaspar.perez@iata.csic.es)) and Carmina Montoliu ([carmina.montoliu@uv.es](mailto:carmina.montoliu@uv.es))

**Contents**

- **Supplementary methods**
  - Assessment of habitual dietary intake
  - DNA extraction and library preparation
  - Sequence processing and bacterial profiling
  - Statistical analyses
  - Supplementary references
- **Supplementary Tables**
  - **Supplementary Table 1.** Biochemical, immunological and HRQoL variables included in the PERMANOVA analysis.
  - **Supplementary Table 2.** Plasma inflammatory variables of healthy controls and patients.
  - **Supplementary Table 3.** SF 36 items compared across groups.
  - **Supplementary Table 4.** Pairwise comparisons of alpha diversity indices.
  - **Supplementary Table 5.** Serum and faecal levels of short-chain fatty acids (SCFA) in healthy controls and patients
- **Supplementary Figures**
  - **Supplementary Figure 1.** Dietary analysis.
  - **Supplementary Figure 2.** Circos plot illustrating significant positive (A) and negative (B) associations among individual features across different biological characteristics.
  - **Supplementary Figure 3.** ROC curves for the biomarker panel.
  - **Supplementary Figure 4.** Taxonomic (A) and functional (B) features associated with fibrosis stage.
  - **Supplementary Figure 5.** Heatmap displaying significant relationships between GMMs (Gut Metabolic Module) and meta-variables, on the x and y-axis respectively.
  - **Supplementary Figure 6.** Comparative box and dot plots analysis of SCFA.

**Supplementary methods**

***Assessment of habitual dietary intake***

A Food Frequency Questionnaire (FFQ) validated for the Spanish population was used to estimate the average dietary intake of the disease groups (MASLD and cirrhosis) [1]. This step was taken because diet has a significant impact on the microbiota's composition, making it essential to document it and thereby reduce variability in this type of study [2]. The FFQ covered 71 different foods, each with nine frequency options, ranging from "never/almost never" to "more than 6 times a day". Subsequently, the average daily consumption was calculated, and the 71 foods were classified into the following categories: whole dairy products, semi-skimmed and skimmed dairy products, eggs, lean meats and cold meats, fatty meats and sausages, white fish and shellfish, blue fish, vegetables, fruits, nuts, beans and legumes, olive oil, other fats, refined cereals, whole grain cereals, industrial confectionery, sugars, alcohol, other foods, and beverages. Following the same methodology as previous studies [3,4], the food categories were used to derive dietary patterns.

***DNA extraction and library preparation***

Approximately 100 mg of stool sample were chemically disrupted with CTAB (cetyltrimethylammonium bromide). Mechanical lysis was performed using 3 µm diameter glass beads and two cycles of 30 s, at a speed of 6 m/s, in FastPrep 24-5G Homogenizer bead beater. To each sample, 40 µL of proteinase K (20mg/mL) and 20 µL of RNAase A (4mg/mL) were added, followed by incubation at 70°C for 10 min. DNA was extracted using an automated magnetic bead-assisted technique (Maxwell RSC Instrument with Maxwell RSC Pure Food GMO and authentication kit; Promega, Spain) according to the manufacturer's protocol.

DNA concentration was quantified using a Qubit 2.0 fluorometer (Life Technology, Carlsbad, CA, USA). The DNA concentration from all samples was adjusted to a final concentration of 5 ng/µL. Extraction controls were included in each round and a pool of these was sequenced together with the samples. In order to compare the efficiency of the extraction with and without RNAlater™ Stabilization Solution (ThermoFisher Scientific; Whaltham, MA, USA), some of the samples were also collected in RNA*later®* and then treated following the procedure described above.

Previously reported primers aiming targeting the V3-V4 variable regions (approximately 459bp) of the 16S rRNA gene were used for amplicon sequencing [5]. Target amplification was performed as described by Illumina library preparation manual [6], and Illumina sequencing adapters and dual-index barcodes from the Nextera XT index kit v2 (FC-131-2001) were incorporated. Libraries were normalized and pooled in equimolar concentrations. Pooled libraries were sequenced on an Illumina MiSeq sequencing system using the MiSeq Reagent Kit V2 500 cycles for paired-end 2x300pb reads (Illumina Reagent Cartridge v3 MS102-3003) with 10% PhiX control. Sequencing was carried out at the Fundación para el Fomento de la Investigación Sanitaria y Biomédica de la Comunitat Valenciana (Fisabio).

***Sequence processing and bacterial profiling***

Sequence quality assessment and preprocessing were performed using the DADA2 (v1.18.0) [7] pipeline within the R (v4.0.3) environment [8] to infer amplicon sequence variants (ASV) from the SILVA (v138. 1) database, with specific modifications applied: primer sequences were trimmed from all reads, and the forward and reverse reads were truncated to lengths of 280 and 265 bases, respectively. Based on the expected size of the 16S rRNA amplicon (459 bp), fragments between 400-428 bp (after trimming) were selected *in silico*. All other parameters were set to default values. The taxonomic, ASV abundance, and sample data were merged into a single object using the phyloseq package (v1.38.0).

All filtering, and statistical analyses were performed using R programming language (v4.1.2) [8] in RStudio (v2021.09.0). The decontam package (v1.14.0) was employed to detect and discard potential contaminants, using the frequency and prevalence (threshold=0.5) methods. Subsequently, a thorough manual assessment of all ASV identified as contaminants was then carried out. The criteria for this assessment included a search of the existing literature to determine whether each identified ASV had been previously identified as a contaminant or whether it was a common species in the human microbiota. ASV confirmed to be contaminants were removed.

Low prevalence ASV with less than 5 counts in 10% of the samples were excluded from the analysis. The ASV counts were transformed to relative abundances for differential abundance analysis, and to central log-ratio (clr) counts for further statistical analyses.

All analyses are based on non-rarefied data. All samples reached saturation in the rarefaction curves and the sequencing depth difference between samples was less than one order of magnitude (17973– 82629).

***Statistical analyses***

A Principal Components Analysis (PCA) using princomp function from the stats package (v 4.1.1) was performed to identify dietary patterns. The process of dietary pattern selection consisted of visual inspection of scree plots in conjunction with eigenvalues (>1) and the interpretability of principal components. Lastly, Dunnett nonparametric tests and Wilcoxon rank sum exact tests subjected to Benjamini-Hochberg (BH) adjustments were used to compare the dietary patterns among groups.

Shannon, Chao1, and Fisher alpha diversity indices were calculated using the R package phyloseq (v1.38.0). Groups were compared using Wilcoxon rank sum exact test implemented in the R package ggpubr (v0.6.0), and *P* values were adjusted for false discovery rate (FDR) using the Benjamini–Hochberg (BH) procedure [9]. Visualization was generated using the packages ggplot2 (v3.4.2) and ggpubr (v0.6.0).

Permutational analysis of variance (PERMANOVA) was performed for non-parametric multivariant statistical testing using adonis function of the vegan package (v2.6.4). We obtained the marginal effects for the predictors stratifying by extraction batch using 999 permutations and used the Euclidean distances for clr-transformed data (ASV) and the Bray-Curtis distances for non-transformed data (GMMs, HRQoL, immunological and biochemical variables) (Supplementary Table 2 indicates which biochemical, immunological and HRQoL variables were included in the analysis). Interaction effect between PHES and age was tested and could be excluded due to lack of significance.

MetadeconfoundR [10] (v0.2.8) was used to assess confounder-aware associations between ASV and GMMs across metadata and detection of confounded covariates. MetadeconfoundR uses a linear model for each feature and the predictors. Further model comparison is done using a Likelihood Ratio Test (LRT) to infer significant associations between features and the metadata variables (FDR < 0.1). The extraction batch was included as a random variable. First, we aimed to identify changes in the analytical parameters and taxonomical (ASV level) and functional changes between disease-control (MASLD vs. control and cirrhosis vs. control) and disease stage to compare patients with cognitive (MCI and MHE) vs without cognitive impairment (NMCI and NMHE). Then, MetadeconfoundR was also employed to identify significant associations between multiple variables from the main six different biological features studied here, including Biochemistry, Immunology, SCFAs, Quality of Life (QoL), Microbiome (Genera and GMMs), and Phenotypic Data. The selection of variables was based on a significance threshold of p ≤ 0.001, ensuring robust associations. Additionally, effect size (Ds) was used as a criterion to classify relationships, with positive associations defined as Ds > 0.4 and negative associations as Ds < -0.4. The filtered data was then visualized using a circos plot, where each feature category was assigned a distinct color. The visualization was generated using the circlize (v0.4.16) package in R.

For identifying variables of interest for further investigation, a PCA was performed utilizing the princomp function from the stats package (v 4.1.1). Additionally, a Pearson's correlation analysis was conducted utilizing the ggpairs function from the GGally package (v 2.1.2) to evaluate and visualize the correlations among SCFA concentrations.

A generalized linear mixed-effects model (GLMM) was employed to evaluate the relationship between SCFA ratios (response variable) and disease subgroups (fixed effect), with pairwise SCFA comparisons included as a random effect. Likelihood ratio tests compared models with and without the subgroup variable to assess its significance. The relative contribution of Group to the variance was determined using the partR2 package (v0.9.1.9000) with bootstrapped marginal R^2^ estimates (1,000 iterations), and results were visualized using forest plots. To identify significant differences marginal means (least-squares means) and pairwise comparisons between subgroups were performed. Model diagnostics, summaries, and ANOVA were also used for more statistical robustness.

Descriptive statistics for each variable were computed, including measures of central tendency (median) and dispersion (interquartile range). These values were reported for each group to provide a summary of the data distribution (Table 1). To evaluate differences between groups, the Wilcoxon rank-sum test was applied adjusting by BH method. For general comparisons, values from the Control group were tested against all other groups (^*^) and specific pairwise comparisons were performed between NMCI vs. MCI and NMHE vs. MHE to address group-specific hypotheses (^α^).

***Predicted functional annotation***

Predicted the functionality of the gut microbiome was obtained using PICRUSt2 (Phylogenetic Investigation of Communities by Reconstruction of Unobserved States 2) algorithm (<https://github.com/picrust/picrust2>) based on ASV counts [11]. PICRUSt2 generates an estimation of the abundance of KEGG Orthologs (KO), predicted metagenomes, and pathways [12,13]. Omixer-rpmR [14] was used to classify predicted KO counts to gut metabolic modules (GMMs), using the coverage cut-off by default to select the combination of KOs that maximize the abundance of each module.

To trace tryptophan (trp) metabolism, the following modules were manually added to the Omixer-rpmR clustered databases based on KEGG modules and the Kaur *et al.* [15] criteria for pathway prediction: kynurenine biosynthesis I (K00453, K00463, K01432, K14263, K07130, K00486, K01556, K00452, K03392, K10217, K23234); melatonin biosynthesis I (K00502, K01593, K00669, K00543); quinolinic acid biosynthesis I (K00453, K00463, K01432, K14263, K07130, K00486, K01556, K00452, K00767, K00969, K06210, K01916, K01950); indole biosynthesis (K01667); indole propionic acid from trp (K13607); tryptamine propionic acid from trp (K01593).

**Supplementary References**

[1] Schröder H, Fitó M, Estruch R, Martínez-González MA, Corella D, Salas-Salvadó J, et al. A Short Screener Is Valid for Assessing Mediterranean Diet Adherence among Older Spanish Men and Women. J Nutr 2011;141:1140–5. <https://doi.org/10.3945/jn.110.135566>

[2] York A. Your microbiome is what you eat. Nat Rev Microbiol 2019;17:721. <https://doi.org/10.1038/s41579-019-0287-1>

[3] Satija A, Hu FB, Bowen L, Bharathi A V., Vaz M, Prabhakaran D, et al. Dietary patterns in India and their association with obesity and central obesity. Public Health Nutr 2015;18:3031–41. <https://doi.org/10.1017/S1368980015000312>

[4] de Souza RJ, Zulyniak MA, Desai D, Shaikh MR, Campbell NC, Lefebvre DL, et al. Harmonization of food-frequency questionnaires and dietary pattern analysis in 4 ethnically diverse birth cohorts. J Nutr. 2016;146:2343–50. <https://doi.org/10.3945/jn.116.236729>

[5] Klindworth A, Pruesse E, Schweer T, Peplies J, Quast C, Horn M, et al. Evaluation of general 16S ribosomal RNA gene PCR primers for classical and next-generation sequencing-based diversity studies. Nucleic Acids Res 2013;41. <https://doi.org/10.1093/nar/gks808>

[6] Illumina Inc. 16S metagenomic sequencing library preparation. Preparing 16S ribosomal RNA gene amplicons for the Illumina MiSeq System. 16S Metagenomic Sequencing Library Preparation Manual 2013. <https://emea.illumina.com/content/dam/illumina-support/documents/documentation/chemistry_documentation/16s/16s-metagenomic-library-prep-guide-15044223-b.pdf> (accessed December 18, 2023).

[7] Callahan BJ, McMurdie PJ, Rosen MJ, Han AW, Johnson AJA, Holmes SP. DADA2: High-resolution sample inference from Illumina amplicon data. Nat Methods 2016;13:581–3. <https://doi.org/10.1038/nmeth.3869>

[8] Team RC. R: A Language and Environment for Statistical Computing R Foundation for Statistical Computing. 2012. <http://www.r-project.org/> (accessed December 18, 2023).

[9] Benjamini Y, Hochberg Y. Controlling the False Discovery Rate: A Practical and Powerful Approach to Multiple Testing. Journal of the Royal Statistical Society: Series B (Methodological) 1995;57:289–300. <https://doi.org/10.1111/j.2517-6161.1995.tb02031.x>

[10] Sofia Forslund, Ulrike Löber, Till Birkner. MetadeconfoundR. R package. <https://doi.org/10.32614/CRAN.package.metadeconfoundR>

[11] Douglas GM, Maffei VJ, Zaneveld JR, Yurgel SN, Brown JR, Taylor CM, et al. PICRUSt2 for prediction of metagenome functions. Nat Biotechnol 2020;38:685–8. <https://doi.org/10.1038/s41587-020-0548-6>

[12] Kanehisa M, Goto S. KEGG: Kyoto Encyclopedia of Genes and Genomes. Nucleic Acids Res. 2000 Jan 1;28(1):27-30. <https://doi.org/10.1093/nar/28.1.27>

[13] Kanehisa M, Furumichi M, Sato Y, Kawashima M, Ishiguro-Watanabe M. KEGG for taxonomy-based analysis of pathways and genomes. Nucleic Acids Res 2023;51:D587–92. <https://doi.org/10.1093/nar/gkac963>

[14] Darzi Y, Falony G, Vieira-Silva S, Raes J. Towards biome-specific analysis of meta-omics data. ISME Journal 2016;10:1025–8. <https://doi.org/10.1038/ismej.2015.188>

[15] Kaur H, Bose C, Mande SS. Tryptophan Metabolism by Gut Microbiome and Gut-Brain-Axis: An in silico Analysis. Front Neurosci 2019;13. <https://doi.org/10.3389/fnins.2019.01365>

**Supplementary Table 1.** Biochemical, immunological and HRQoL variables included in the PERMANOVA analysis. HDL, high-density lipoprotein; LDL, low-density lipoprotein; TG, triglycerides; AST, aspartate transaminase; ALT, alanine transaminase; GGT, gamma-glutamyltransferase; ALP, alkaline phosphatase; HB, hemoglobin; QI, Quick index; INR, international normalized ratio; IL, interleukin; CCL20, C‐C motif chemokine ligand 20; TNF-α, tumor necrosis factor alpha; CXCL13, C‐X‐C motif chemokine ligand 13.

| **Kind of variable** | **Variables included** |
| --- | --- |
| Biochemical variables | Glucose (mg/dL), Creatinine (mg/dL), Cholesterol (mg/dL), HDL (mg/dL), LDL (mg/dL), TG (mg/dL), total proteins (g/dL), albumin (g/dL), bilirrubin (mg/dL), AST (U/L), ALT (U/L), GGT (U/L), ALP (mU/mL), leucocytes (x10^9^/L), HB (g/dL), platelets (x10^9^/L), QI (%), INR. |
| Immunological variables (pg/mL) | IL-6, IL-18, IL-23, IL-21, IL-13, CCL20, TNF-α, CXCL13, IL-22, IL-4. |
| HRQoL variables | Physical functioning, physical role, emotional role, vitality, mental health, social functioning, bodily pain, general health, health transition, overall score. |

**Supplementary Table 2.** Plasma inflammatory variables of healthy controls and patients. Values are the median (1st quartile, 3rd quartile). Values significantly different from those in the controls are indicated by an asterisk (*) and from those in NMCI vs MCI or MHE vs. NMHE patients by α (*/α p < 0.05; **/αα p < 0.01; ***/ααα p < 0.001). MASLD, metabolic dysfunction-associated steatotic liver disease; NMCI, non-mild cognitive impairment; MCI, mild cognitive impairment; NMHE, patients without minimal hepatic encephalopathy; MHE, patients with minimal hepatic encephalopathy; IL, interleukin; CCL20, C‐C motif chemokine ligand 20; TNF-α, tumor necrosis factor alpha; CXCL13, C‐X‐C motif chemokine ligand 13; BDNF, Brain Derived Neurotrophic Factor.

| **Inflammatory variables (pg/mL)** | **Control** | **MASLD patients** | | **Cirrhosis patients** | |
| --- | --- | --- | --- | --- | --- |
|  |  | **NMCI** | **MCI** | **NMHE** | **MHE** |
| **IL-6** | 0.67  (0.42, 1.31) | 2.49  (1.61, 3.18) *** | 5.54  (4.56, 6.16) ***^/ ααα^ | 2  (1.27, 2.97) *** | 7.22 (5.62, 8.42) ***^/ααα^ |
| **IL-18** | 100.56  (72.31, 118.04) | 191.12  (144.76, 236.17) *** | 251.51  (197.65, 344.88) ***^/ α^ | 173.93  (126.3, 209.7) *** | 582.15 (484.38, 610.4) ***^/ααα^ |
| **IL-23** | 5.6  (2.83, 9.1) | 6.8  (3.69, 18.47) | 71.75  (60.83, 105.5) ***^/ααα^ | 10.1  (5.1, 18.1) * | 20.1 (14.1, 34.85) ***^/αα^ |
| **IL-21** | 122.12  (106.25, 138.88) | 12.02  (7.38, 19.34) *** | 95.23  (73.36, 114.34) ^ααα^ | 131  (108.5, 192.75) | 1307.88  (934, 1740.25) ***^/ααα^ |
| **IL-13** | 1.56  (1.33, 2.13) | 2  (1.69, 3) * | 16.68  (15.41, 25.36) ***^/ααα^ | 1.84 (1.55, 3.53) * | 3.42  (2.05, 10.24) ** |
| **CCL20** | 6.82  (5.55, 9.52) | 2.44  (2.09, 3.42) *** | 67.42  (56.52, 87.12) ***^/ααα^ | 31.32  (18.93, 58.37) *** | 104.56 (90.34, 133.1) ***^/ααα^ |
| **TNF-α** | 1.33  (1.19, 1.46) | 5.75  (4.93, 7.05) *** | 5.87  (4.84, 9.37) *** | 1.82 (1.51, 1.92) *** | 3.39 (2.57, 4.12) ***^/ααα^ |
| **CX3CL1** | 1096.01  (570.1, 2056.4) | 2725.3  (2231.64, 3516.83) *** | 3793  (2418.77, 4547.69) *** | 2756.02  (2281.16, 3435.35) *** | 3296.25 (2952.22, 4066.32) ***^/α^ |
| **CXCL13** | 57.85  (50.46, 66.11) | 40.3  (21.02, 61) * | 89  (24.98, 135.91) | 118.72  (88.28, 153.72) *** | 231.11 (193.07, 261.65) ***^/ααα^ |
| **IL-22** | 62.86  (54.03, 70.4) | 156.71  (149.57, 191) *** | 443.86  (318.14, 518.86) ***^/ααα^ | 12.71  (5.65, 33) *** | 134.1  (77.79, 183.34) ***^/ααα^ |
| **IL-4** | 1.59  (1.52, 1.7) | 64.03  (56.03, 70.37) *** | 70.7  (65.87, 75.87) *** | 1.7  (1.62, 1.82) * | 1.71  (1.6, 1.91) * |
| **BDNF** | 636.19  (311.5, 778.04) | 574.7  (365.16, 688.73) | 408.39  (274.85, 489.33) ^α^ | 206.49  (127.68, 307.97) * | 314.84  (167.71, 457.71) |

**Supplementary Table 3. SF 36 items compared across groups.** Values are the median (1st quartile, 3rd quartile). Values significantly different from those in the controls are indicated by an asterisk (*) and from those in NMCI vs MCI or MHE vs. NMHE patients by α (*^/α^ p < 0.05; ^αα^ p < 0.01). MASLD, metabolic dysfunction-associated steatotic liver disease; NMCI, non-mild cognitive impairment; MCI, mild cognitive impairment; NMHE, patients without minimal hepatic encephalopathy; MHE, patients with minimal hepatic encephalopathy.

| **SF 36 items** | **Control** | **MASLD patients** | | **Cirrhosis patients** | |
| --- | --- | --- | --- | --- | --- |
|  |  | **NMCI** | **MCI** | **NMHE** | **MHE** |
| **Physical function** | 90 (85, 97.5) | 90 (80, 95) | 75 (65, 86.2) | 77.5 (55, 86.2) | 52.5 (45, 77.5) |
| **Physical role** | 100 (100, 100) | 100 (75, 100) | 100 (100, 100) | 75 (18.75, 100) | 25 (0, 50)* |
| **Emotional role** | 100 (100, 100) | 100 (66.7, 100) | 100 (58.3, 100) | 100 (66.7, 100) | 50 (5.6, 91.9)^α^ |
| **Vitality** | 75 (50, 85) | 70 (45, 85) | 67.5 (40, 71.2) | 50 (38.7, 72.5) | 47.5 (20, 63.7) |
| **Mental health** | 84 (64, 90) | 80 (68, 84) | 74 (64, 93) | 72 (60, 90.5) | 48 (40, 57)^αα^ |
| **Social function** | 100 (81.2 100) | 100 (87.5, 100) | 93.7 (62.9, 100) | 83.7 (71.9, 100) | 68.7 (34.4, 78.5) |
| **Pain** | 80 (68.7, 95) | 80 (45, 90) | 75 (41, 85) | 67.5 (45, 90) | 57.5 (25, 75) |
| **General health** | 75 (72.5, 80) | 60 (50, 65) | 42.5 (38.7, 51.2)^α^ | 57.5 (43.7, 70) | 52.5 (35.2, 73.7) |
| **Health transition** | 50 (50, 50) | 50 (50, 75) | 50 (25, 56.2) | 50 (50, 75) | 50 (25, 50) |
| **Final score SF36** | 85.6 (71.3, 87.1) | 79.8 (67.3, 88.2) | 78.2 (53.5, 80.2) | 65.3 (53.8, 82.3) | 49.5 (25.8, 68.3) |

| **index** | **group1** | **group2** | **n1** | **n2** | **statistic** | **p** | **p.adj** | **p.adj.signif** | **y.position** | **xmin** | **xmax** |
| --- | --- | --- | --- | --- | --- | --- | --- | --- | --- | --- | --- |
| Shannon | Control | NMCI | 18 | 33 | 388 | 0.074 | 0.154 | ns | 4.90736 | 1 | 2 |
| Shannon | Control | MCI | 18 | 11 | 136 | 0.102 | 0.17 | ns | 4.96442667 | 1 | 3 |
| Shannon | Control | NMHE | 18 | 37 | 503 | 0.002 | 0.02 | * | 5.02149333 | 1 | 4 |
| Shannon | Control | MHE | 18 | 14 | 183 | 0.03 | 0.15 | ns | 5.07856 | 1 | 5 |
| Shannon | NMCI | MCI | 33 | 11 | 166 | 0.689 | 0.76555556 | ns | 5.13562667 | 2 | 3 |
| Shannon | NMCI | NMHE | 33 | 37 | 777 | 0.05 | 0.154 | ns | 5.19269333 | 2 | 4 |
| Shannon | NMCI | MHE | 33 | 14 | 287 | 0.199 | 0.28428571 | ns | 5.24976 | 2 | 5 |
| Shannon | MCI | NMHE | 11 | 37 | 276 | 0.077 | 0.154 | ns | 5.30682667 | 3 | 4 |
| Shannon | MCI | MHE | 11 | 14 | 99 | 0.244 | 0.305 | ns | 5.36389333 | 3 | 5 |
| Shannon | NMHE | MHE | 37 | 14 | 264 | 0.925 | 0.925 | ns | 5.42096 | 4 | 5 |
| Chao1 | Control | NMCI | 18 | 33 | 457.5 | 0.002 | 0.00666667 | ** | 380.44 | 1 | 2 |
| Chao1 | Control | MCI | 18 | 11 | 157.5 | 0.009 | 0.0225 | * | 392.873333 | 1 | 3 |
| Chao1 | Control | NMHE | 18 | 37 | 508 | 0.002 | 0.00666667 | ** | 405.306667 | 1 | 4 |
| Chao1 | Control | MHE | 18 | 14 | 216 | 0.000673 | 0.00666667 | ** | 417.74 | 1 | 5 |
| Chao1 | NMCI | MCI | 33 | 11 | 171 | 0.786 | 0.786 | ns | 430.173333 | 2 | 3 |
| Chao1 | NMCI | NMHE | 33 | 37 | 692.5 | 0.338 | 0.4275 | ns | 442.606667 | 2 | 4 |
| Chao1 | NMCI | MHE | 33 | 14 | 296 | 0.133 | 0.266 | ns | 455.04 | 2 | 5 |
| Chao1 | MCI | NMHE | 11 | 37 | 236 | 0.432 | 0.48 | ns | 467.473333 | 3 | 4 |
| Chao1 | MCI | MHE | 11 | 14 | 97 | 0.286 | 0.4275 | ns | 479.906667 | 3 | 5 |
| Chao1 | NMHE | MHE | 37 | 14 | 304.5 | 0.342 | 0.4275 | ns | 492.34 | 4 | 5 |
| Fisher | Control | NMCI | 18 | 33 | 446 | 0.003 | 0.01 | ** | 53.49816 | 1 | 2 |
| Fisher | Control | MCI | 18 | 11 | 154 | 0.013 | 0.0325 | * | 55.3038933 | 1 | 3 |
| Fisher | Control | NMHE | 18 | 37 | 505 | 0.002 | 0.01 | ** | 57.1096267 | 1 | 4 |
| Fisher | Control | MHE | 18 | 14 | 209 | 0.001 | 0.01 | ** | 58.91536 | 1 | 5 |
| Fisher | NMCI | MCI | 33 | 11 | 172 | 0.81 | 0.81 | ns | 60.7210933 | 2 | 3 |
| Fisher | NMCI | NMHE | 33 | 37 | 709 | 0.251 | 0.38142857 | ns | 62.5268267 | 2 | 4 |
| Fisher | NMCI | MHE | 33 | 14 | 298 | 0.123 | 0.246 | ns | 64.33256 | 2 | 5 |
| Fisher | MCI | NMHE | 11 | 37 | 242 | 0.356 | 0.40333333 | ns | 66.1382933 | 3 | 4 |
| Fisher | MCI | MHE | 11 | 14 | 98 | 0.267 | 0.38142857 | ns | 67.9440267 | 3 | 5 |
| Fisher | NMHE | MHE | 37 | 14 | 303 | 0.363 | 0.40333333 | ns | 69.74976 | 4 | 5 |

**Supplementary Table 4.** Pairwise comparisons of alpha diversity indices among subgroups using the non-parametric Wilcoxon test with Benjamini-Hochberg adjusted p-values.

**Supplementary Table 5. Faecal levels of short-chain fatty acids (SCFA) in healthy controls and patients.** Values are the median (1st quartile, 3rd quartile). MASLD, metabolic dysfunction-associated steatotic liver disease; NMCI, non-mild cognitive impairment; MCI, mild cognitive impairment; NMHE, patients without minimal hepatic encephalopathy; MHE, patients with minimal hepatic encephalopathy.

| **SCFA in faeces** | **Control** | **MASLD patients** | | **Cirrhosis patients** | |
| --- | --- | --- | --- | --- | --- |
|  |  | **NMCI** | **MCI** | **NMHE** | **MHE** |
| Acetic acid | 3.46 (1.98, 4.97) | 4 (3.05, 5.86) | 7.12 (5.57, 7.72) | 2.44 (1.31, 4.03) | 3.37 (2.77, 4.76) |
| Propionic acid | 0.66 (0.29, 0.76) | 0.75 (0.44, 1.23) | 0.98 (0.96, 1.88) | 0.63 (0.23, 0.85) | 0.72 (0.6, 0.95) |
| Butyric acid | 0.86 (0.51, 1.6) | 1.25 (0.94, 1.56) | 1.36 (1.16, 1.54) | 0.96 (0.41, 1.31) | 1.04 (0.85, 1.23) |
| Isobutyric acid | 0.06 (0.03, 0.13) | 0.11 (0.07, 0.23) | 0.13 (0.09, 0.15) | 0.07 (0.04, 0.13) | 0.1 (0.09, 0.13) |
| Caproic acid | 0.02 (0.02, 0.04) | 0.03 (0.02, 0.06) | 0.05 (0.04, 0.07) | 0.03 (0.02, 0.06) | 0.02 (0.02, 0.03) |
| Valeric acid | 0.14 (0.1, 0.19) | 0.18 (0.13, 0.27) | 0.28 (0.23, 0.31)/ | 0.2 (0.16, 0.25) | 0.16 (0.11, 0.18) |
| Isovaleric acid | 0.12 (0.05, 0.17) | 0.12 (0.09, 0.21) | 0.14 (0.12, 0.21) | 0.13 (0.11, 0.2) | 0.14 (0.13, 0.2) |
| 2-Methylbutyric acid | 0.05 (0.02, 0.09) | 0.06 (0.04, 0.12) | 0.1 (0.07, 0.11) | 0.06 (0.03, 0.12) | 0.08 (0.07, 0.11) |

**Fig. S1.** Dietary analysis. (A) Principal Components Analysis clustering samples by group, with vectors representing specific aliment groups from the Food Frequency Questionnaire (FFQ). (B) Radar chart illustrating the presence or absence of aliment groups in the four food patterns derived from PCA. (C) Violin plots of each food pattern by group, with no significant statistical differences observed between groups.

**Fig. S2. Circos plot illustrating significant positive (A) and negative (B) associations among individual features across different biological characteristics** (biochemistry, immunology, SCFAs, quality of life (QoL), both taxonomic and functional microbiome profiles and phenotypic data). Each line between two features shows a significant association (Mann-Whitney U/Spearman Correlation test, FDR-corrected p-values <0.001). The line thickness indicates the effect size (Cliff's Delta/Spearman's rho, only correlations with a minimum effect size > 0.4 for positive associations and a minimum effect size < -0.4 for negative associations were plotted) of the respective association. The outer ring segments show corresponding feature biological domains (color) and number/strength of associations for each feature (width). HDL, High-Density Lipoprotein; ALB, albumin; TG, triglycerides; HB, hemoglobin; GGT, Gamma-Glutamyl Transferase; GABA, gamma-aminobutyric acid; IL, Interleukin; CXCL13, Chemokine (C-X-C motif) ligand 13; CCL20, Chemokine (C-C motif) ligand 20; ARG, arginine; TNFα, tumor necrosis factor, Glu, glutamic acid; VA, valeric acid; IVA, isovaleric acid; IBA, isobutyric acid; CA, caproic acid; 2MBA: 2-Methylbutyric acid; NMCI (Non-Mild Cognitive Impairment); MCI (Mild Cognitive Impairment); NMHE (Non-Minimal Hepatic Encephalopathy); MHE (Minimal Hepatic Encephalopathy).


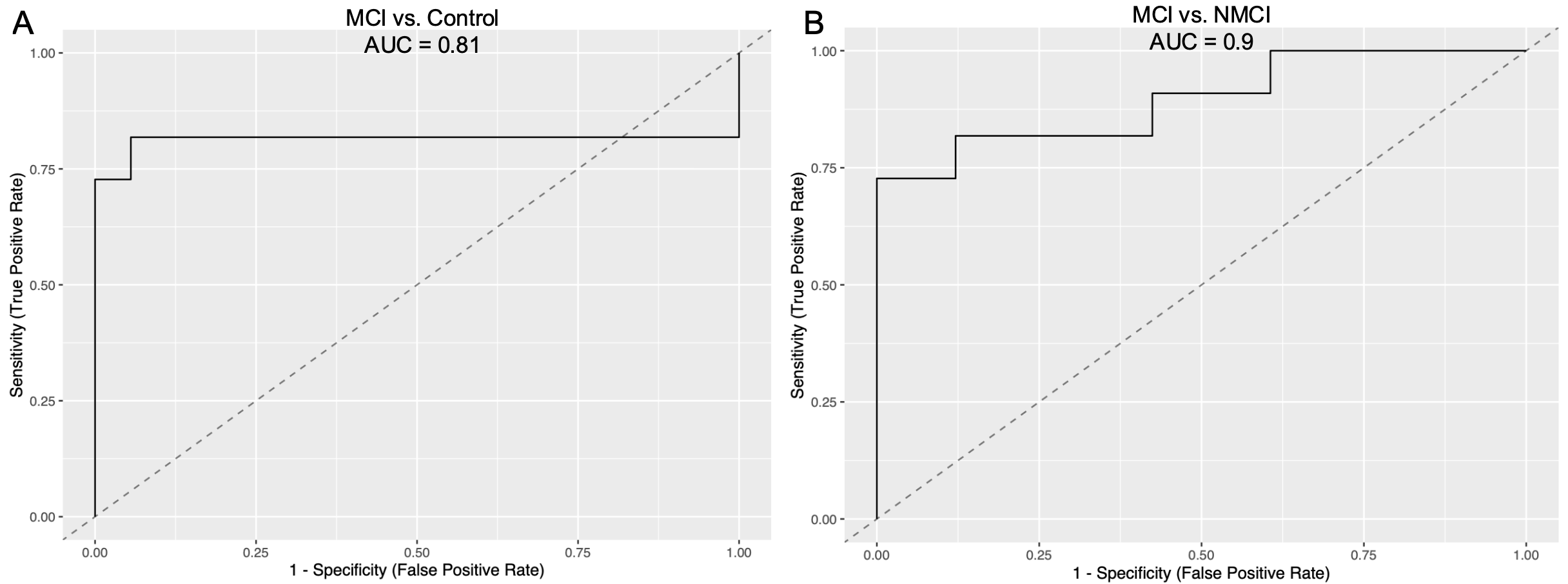


**Fig. S3.** ROC curves for the biomarker panel (*St. mutans* and *A. histaminiformans*) models and associated AUC values. (A) MASLD patients with Mild Cognitive Impairment (MCI) vs. Control (B) MASLD patients with MCI vs. MASLD patients without MCI.

**Fig. S4.** Taxonomic (A) and functional (B) features associated with fibrosis stage. The colour scale and size illustrates the effect size, and the y-axis colour scale on the left indicates the family to which each ASV belongs (indicated in the legend). Significance is denoted by black asterisks based on FDR-adjusted p-values, with grey circles representing associations that, although significant, are confounded. Significance levels: p<0.05*/º, p<0.01**/ºº,p<0.001***/ººº.

**Fig. S5.** Heatmap displaying significant relationships between GMMs (Gut Metabolic Module) and meta-variables, on the x and y-axis respectively. The colour scale and size illustrates the effect size. Significance is denoted by black asterisks based on FDR-adjusted p-values, with grey circles representing associations that, although significant, are confounded. Significance levels: p<0.05*/º, p<0.01**/ºº,p<0.001***/ººº. HB, hemoglobin; ARG, arginine; AST aspartate transaminase; ALT, alanine transaminase; IL, Interleukin; CXCL13, Chemokine (C-X-C motif) ligand 13; CCL20, Chemokine (C-C motif) ligand 20; TNFα, tumor necrosis factor; PA, propionic acid; CA, caproic acid.

**
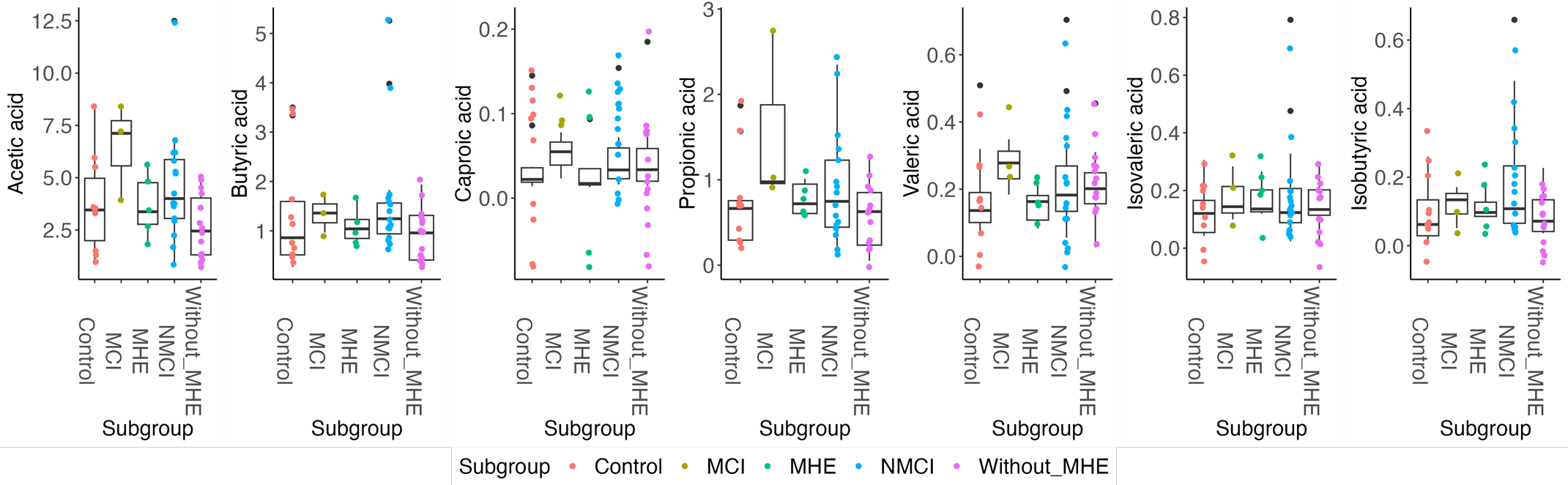
**

**Fig. S6.** Comparative box and dot plots analysis of SCFA between different groups (n=8 per group). No asterisk is marked because of no statistical differences.
